# Comparative transcriptome analysis reveal gene regulation of dormancy release in *Cardiocrinum giganteum* seeds induced by temperature

**DOI:** 10.3389/fpls.2025.1591781

**Published:** 2025-07-17

**Authors:** Yefang Li, Xuejiao Li, Fengrong Li, Lele Wang, Hongling Li, Yan Zhao, Wenling Guan

**Affiliations:** ^1^ College of Landscape and Horticulture, Yunnan Agricultural University, Kunming, China; ^2^ The Graduate School, Kunming Medical University, Kunming, China

**Keywords:** *Cardiocrinum giganteum*, seed dormancy, alternating temperature stratification, transcriptome, metabolism pathway

## Abstract

The *Cardiocrinum giganteum* is a bulbous plant with extremely high ornamental and economic values. The study revealed that seeds require an extended period of variable temperature stratification treatment to overcome dormancy and initiate germination, yet the molecular mechanisms underlying embryo dormancy release remain unclear. In this research, transcriptome profiles at different germination stages of seeds subjected to variable temperature stratification were systematically analyzed and compared, while the embryo length of corresponding seed samples was quantitatively measured. The results demonstrated that within the initial 60 days of stratification, the embryo scarcely grew. After 90 days of stratification, the embryo elongated conspicuously, and germination initiated at 130 days of stratification. The transcriptome sequencing outcomes demonstrated that the differentially expressed genes (DEGs) identified in the three comparative groups were predominantly associated with plant hormone signal transduction, carbohydrate metabolic pathways, and phenylpropanoid biosynthesis metabolic pathways. Notably, genes associated with auxin, abscisic acid (ABA), brassinosteroid (BR), ethylene, and gibberellin signaling pathways were significantly upregulated during the stratification period from 30 d to 60 d, while these genes exhibited varying degrees of significant differential expression from 90 d to 130 d. Multiple key enzymes in carbohydrate metabolic pathways exhibited marked upregulation after 90d of stratification. Notably, β-glucosidase (*BGLU*) genes associated with polysaccharide hydrolysis (Cluster-62345.33620, Cluster-62345.31435, and Cluster-62345.35688) showed 6.68-, 5.08-, and 6.85-fold upregulation, respectively, at 130 d of stratification. Concurrently, the glycolytic pathway was upregulated throughout the process. The majority of genes involved in phenylpropanoid biosynthesis, particularly those encoding peroxidases, were activated during stratification. The reliability and accuracy of 10 genes closely associated with *C. giganteum* seed germination were validated using RT-qPCR. The results demonstrated that plant hormone signal transduction, carbohydrate metabolism pathways, and phenylpropanoid biosynthesis collectively participate in the post-maturation development and germination processes of the embryo. The potential roles of certain genes in these developmental and germination stages require further investigation. These findings provide novel insights into the transcriptional regulatory mechanisms underlying dormancy release in *C. giganteum* seeds. The candidate genes identified in this study warrant functional characterization and may contribute to advancing the understanding of seed dormancy and germination in plants.

## 1 Introduction

Seed dormancy is a trait formed by plants during their long-term phylogenetic development for adapting to the environment and perpetuating their existence, possessing universal ecological significance (Baskin and Baskin, 2014). Nevertheless, from the viewpoint of agricultural production, seed dormancy frequently constitutes a significant impediment to seedling production and breeding efforts. Therefore, studies on seed dormancy and the mechanisms of dormancy release are of paramount importance for agricultural production. The development condition of the embryo at seed maturity is closely associated with seed dormancy. For seeds featuring morphophysiological dormancy (MPD), the embryo is not fully developed when the seeds are disseminated and has to undergo a considerable period of morphological and physiological after-ripening before germination (Hilhorst et al., 2010; Baskin and Baskin, 2014; Klupczyńska and Pawłowski, 2021), and thus often has an extended dormancy period, posing significant challenges to seed propagation. Hence, researching the mechanisms of post-maturation development of plant embryos and their regulatory techniques holds great significance for the production of plant seedlings.

Temperature ranks among the most crucial environmental factors that impact the dormancy and germination of plant seeds. Once seeds sense environmental temperature signals, they further trigger the interaction of endogenous hormones, thereby initiating the physiological process of seed germination (Chen et al., 2020; Klupczyńska and Pawłowski, 2021; Quan et al., 2025). Dormant seeds are typically categorized into five types: physiological dormancy, morphological dormancy, morphophysiological dormancy, physical dormancy (PY), and combinational dormancy (PY + PD) (Baskin and Baskin, 2004). Among these, physiological dormancy is the most prevalent type. Stratification under different temperature conditions is the most effective approach for breaking physiological dormancy (Baskin and Baskin, 2004; Chen et al., 2020; Yang et al., 2020). However, the molecular mechanism through which temperature alleviates seed dormancy remains poorly understood.

The genus *Cardiocrinum* is conspicuously differentiated from the genus *Lilium* by its tall plants and heart-shaped leaves with reticulate venation (Ma et al., 2022). This genus encompasses three species: *C. giganteum*, *C. cathayanum*, and *C. cordatum*, among which the former two are native to China. *C. giganteum* (Wall.) Makino, with its tall and upright plants, large leaves, and white and fragrant flowers, possesses extremely high ornamental value and has gained the reputation of “Prince of Lilies” in Europe (Zhao et al., 2025). The seeds of *C. giganteum* have medicinal value and are employed to treat ailments such as cough and asthma (Li et al., 2010; Shou et al., 2018). The bulbs are abundant in nutrients like starch, crude fiber, and mineral elements, and are frequently consumed as wild vegetables (Guan et al., 2011).

Available data indicate that the seeds of all three species in the genus *Cardiocrinum* possess morphophysiological dormancy (Sun et al., 2000; Kondo et al., 2006; Guan et al., 2010; Phartyal et al., 2012), and they require a lengthy dormant period before germination. Our previous studies discovered that the complex variable temperature stratification from high to low temperatures in the laboratory could effectively facilitate the post-maturation development of the embryo of *C. giganteum* and relieve seed dormancy, shortening the seed dormancy period from 18 months to approximately 5 months (Guan et al., 2010; Li et al., 2020), which suggests that temperature is the main factor influencing embryo dormancy. Although we have carried out a preliminary exploration of the physiological mechanism of dormancy release in *C. giganteum* seeds, the molecular mechanism of seed dormancy release is still unknown. Based on this, we selected several crucial time points during the stratification treatment of *C. giganteum* seeds for transcriptome analysis, in the hope of providing a basis for exploring the molecular mechanism of seed germination.

## 2 Materials and methods

### 2.1 Plant materials and seed treatments

Eighty mature capsules were collected from ten plants in a population of *C. giganteum* growing naturally in a moist subtropical evergreen broad leaved forest woodland at an altitude of 2200 m a.s.l. in the Cangshan Mountain of Yunnan Province, China, on 9 November 2020. The capsules harvested from different plants were combined and were put into non-woven fabric envelopes. The capsules arrived at Yunnan Agricultural University, Kunming, Yunnan, China on 10 November 2020 and were allowed to dry naturally in a laboratory (approximately 20°C) for five days, during which time the capsules dehisced. Then, seeds were collected by hand from the opened capsules and were spread onto porcelain dishes and allowed to dry naturally again at ambient room temperature (approximately 20°C) for three days. Undeveloped seeds were discarded. Only visibly well-developed dry seeds were put into plastic envelopes and stored in a drying basin with silica gel at 4°C until used in germination studies.

### 2.2 Alternating temperature stratification experiments in the laboratory

For all experiments in the laboratory, seeds were placed in 90-mm-diameter ×10-mm deep plastic Petri dishes on two sheets of qualitative filter paper moistened with distilled water. Petri dishes were sealed with parafilm to retard water loss during incubation. The daily photoperiod was 12 hours in both constant and daily alternating temperature regimes. In the alternating temperature regimes, high temperature was given for 12 hours in light each day and low temperature for 12 hours in darkness. Seeds incubated at 5°C were kept in constant darkness. The light source was cool white fluorescent tubes, and photon irradiance (400–700 nm) at seed level was 10–15 µmol m^-2^ S^-1^. Seeds were examined at intervals of 15–20 days. In several laboratory experiments, arrows (→) indicate when seeds were moved to the next temperature regime in the sequence. At each observation, seeds with an emerged radicle were recorded and then removed from the dishes. Water was added to dishes as needed to keep the filter paper moist.

### 2.3 Temperature dependence of embryo growth and of seed germination

On 15 November 2020, three Petri dishes containing 50 seeds each were placed at the temperature regimes of 25 / 15°C (60 d) → 15 / 5°C (60 d) → 5°C (10 d). Ten seeds were chosen at random and removed from each of the three dishes in each temperature treatment at 30 day intervals and lengths of the 10 embryos measured as previously described.

### 2.4 Statistical analyses of embryo growth and of seed germination

Statistical analyses were carried out using Microsoft Excel 2010 and SPSS 22.0. Means and standard errors were calculated for embryo length and for radicle and cotyledon emergence. Percent value of embryo length and final cumulative percentage of radicle and of cotyledon emergence for different treatments were analyzed using either t-test or one-way ANOVA followed by the Tukey test (*P*<0.05), if ANOVA showed significant differences. Final percentage values for radicle and cotyledon emergence were arcsine square-root transformed for normal distribution before statistical analysis; non-transformed data are shown in the figures.

### 2.5 RNA sample preparation

Seed samples (0.2g per sample) collected from stratification treatment at 0 d、30 d、60 d、90 d、120 d and 130 d, respectively, were used as experimental materials. The collected seed samples were immediately frozen in liquid nitrogen and stored at −80°C for transcriptome analysis and physiological indicator determination. Those seed samples collected from the six stages were used for transcriptome analysis. All seed samples consisted of three biological replicates at each germination stage.

### 2.6 RNA extraction and high-throughput sequencing

After extracting RNA from qualified samples, a total amount of 1.5 µg RNA per sample was used as input material for the RNA sample preparations. Sequencing libraries were generated using NEBNext^®^ Ultra™ RNA Library Prep Kit for Illumina^®^ (NEB, USA) following manufacturer’s recommendations and index codes were added to attribute sequences to each sample. The clustering of the index-coded samples was performed on a cBot Cluster Generation System using TruSeq PE Cluster Kit v3-cBot-HS (Illumia) according to the manufacturer’s instructions. After cluster generation, the library preparations were sequenced on an Illumina Hiseq platform and paired-end reads were generated. Transcriptome assembly was accomplished based on the left.fq and right.fq using Trinity with min_kmer_cov set to 2 by default and all other parameters set default.

### 2.7 Gene functional annotation and differential expression analysis

Gene function was annotated based on the following databases: Nr (NCBI non-redundant protein sequences); Nt (NCBI non-redundant nucleotide sequences), Pfam (Protein family), KOG/COG (Clusters of Orthologous Groups of proteins), Swiss-Prot (A manually annotated and reviewed protein sequence database), KO (KEGG Ortholog database), GO (Gene Ontology). Genes with an adjusted P-value <0.05 found by DESeq were assigned as differentially expressed. Differential expression analysis of two samples was performed using the DEGseq (Wang et al., 2010) R package. Pvalue was adjusted using q value (Storey, 2003). qvalue<0.005 & |log2(foldchange)|>1 was set as the threshold for significantly differential expression.

### 2.8 qRT−PCR validation

Total RNA was extracted from *C. giganteum* seeds at different stages of variable temperature stratification using a Total RNA Extraction Kit. Reverse transcription was performed following the manufacturer’s instructions (Nuoweizhiyuan Biotechnology Co., Ltd., Beijing, China). The hormone signal transduction pathways in *Arabidopsis thaliana* were curated using the Plant Metabolic Network (PMN, https://www.plantcyc.org/). Homologous genes in *C. giganteum* were identified through local BLAST alignment of *Arabidopsis*-related gene sequences, complemented by domain analysis using the program and sequence similarity assessments. Gene-specific primers for qRT-PCR were designed using the NCBI online primer design tool. Expression levels of hormone signal transduction pathway genes in *C. giganteum* under variable temperature stratification treatment were quantified via qRT-PCR. The relative expression levels were calculated using the 2−ΔΔCT method, and statistical significance of the data was evaluated by independent t-tests in SPSS software (version XX; IBM Corp.).

## 3 Result

### 3.1 Effect of temperature on embryo growth and radicle emergence

**Figure 1:**
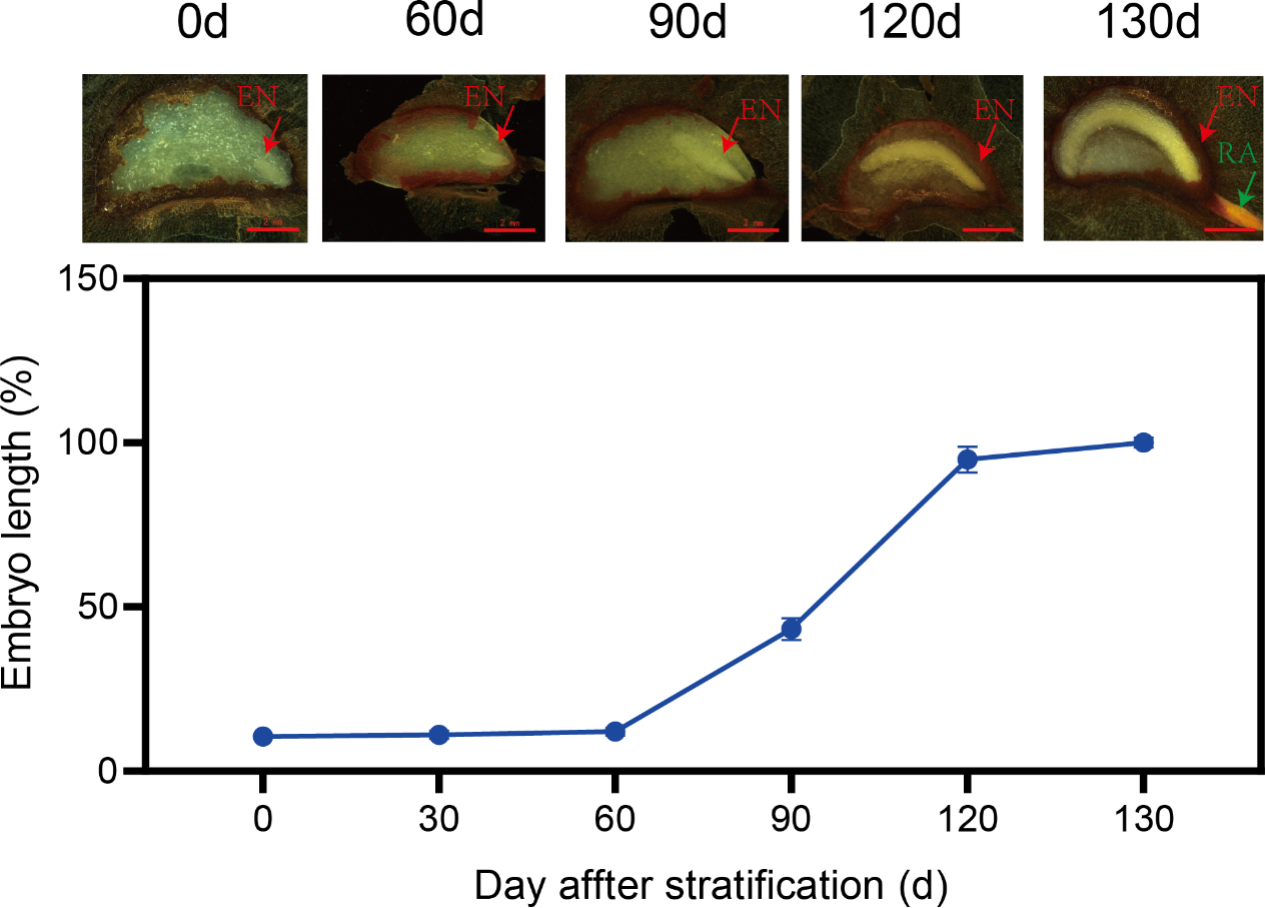
Growth dynamics of *Cardiocrinum giganteum* embryos. The red arrow refers to the embryo (EN), and the green arrow refers to the radicle (RA).

In the temperature-sequence 25 / 15°C (60 d) →15 / 5°C (60 d) → 5°C (60 d), embryos hardly grew during the first temperature regime (i.e., 25 / 15°C) but began to grow rapidly in the second regime (i.e., 15 / 5°C), reaching 43.3% of their full length by 90 d, and reaching 94.9% of their full length by the end of the second regime (120 d). Embryos continued growing rapidly in the third regime (i.e., 5°C), and they became fully elongated by 130 d, by which time radicles had emerged from 21.3% of the seeds (**Figure 1**).

### 3.2 The results of transcriptome sequencing

To comprehensively investigate gene expression dynamics and associated metabolic regulatory pathways during seed development and germination in *C. giganteum*, RNA-seq was performed using the Illumina HiSeq 4000 platform. Sequencing targeted samples collected at distinct temporal phases of variable temperature stratification. Given the exceptionally large genome size of *C. giganteum*, we employed high-depth sequencing (≥8 Gb per sample) to ensure robust transcriptome coverage. A total of 166 Gb of high-quality data was generated across 18 biological replicates, yielding 1,107,551,906 clean reads. Individual samples averaged 9.2 Gb of sequencing data and 61,530,661 clean reads, providing sufficient resolution for downstream analyses of low-abundance transcripts.

**Figure 2:**
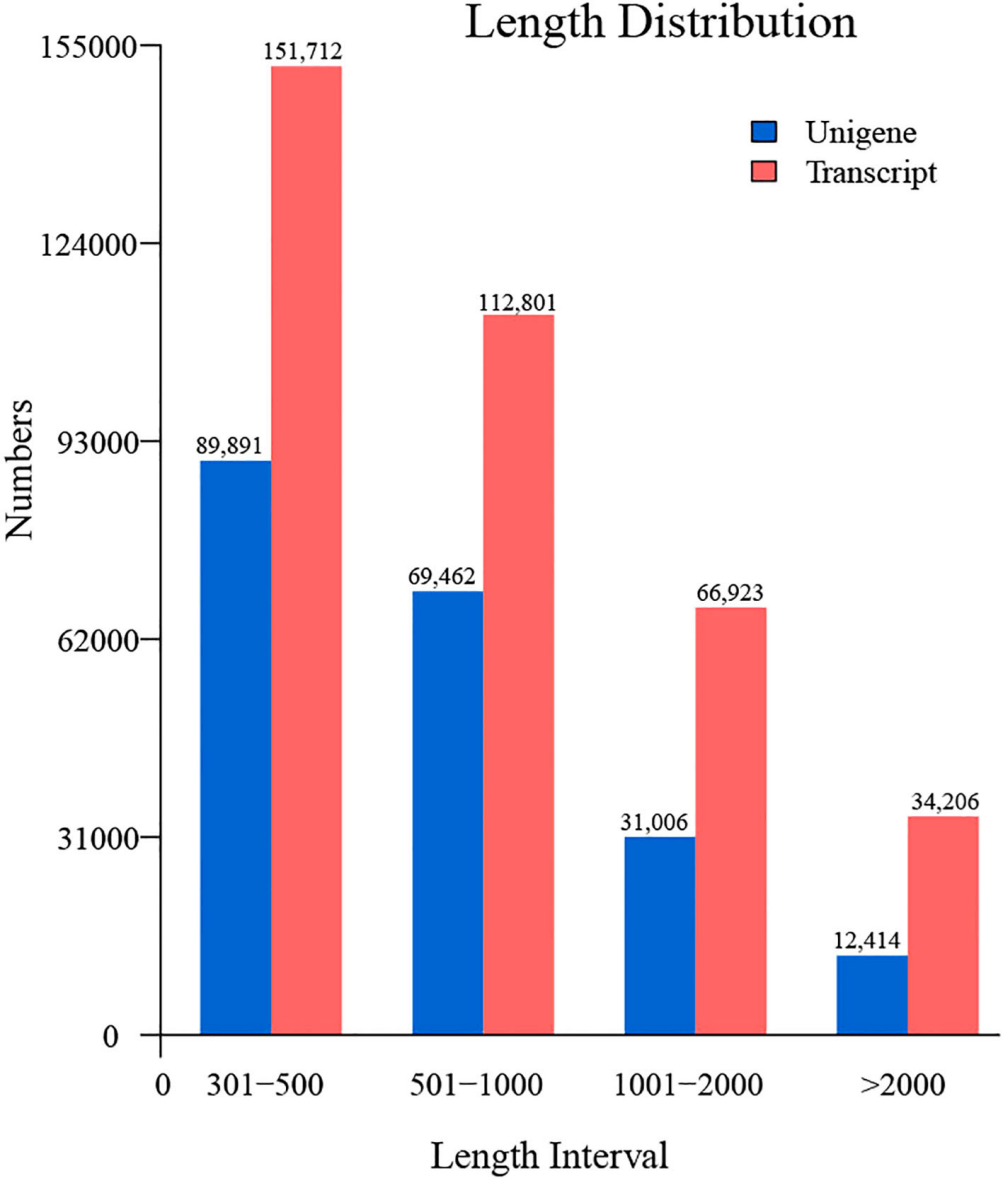
Length distribution profiling of unigenes and transcripts in the *C. giganteum* transcriptome assembly.

**Figure 3:**
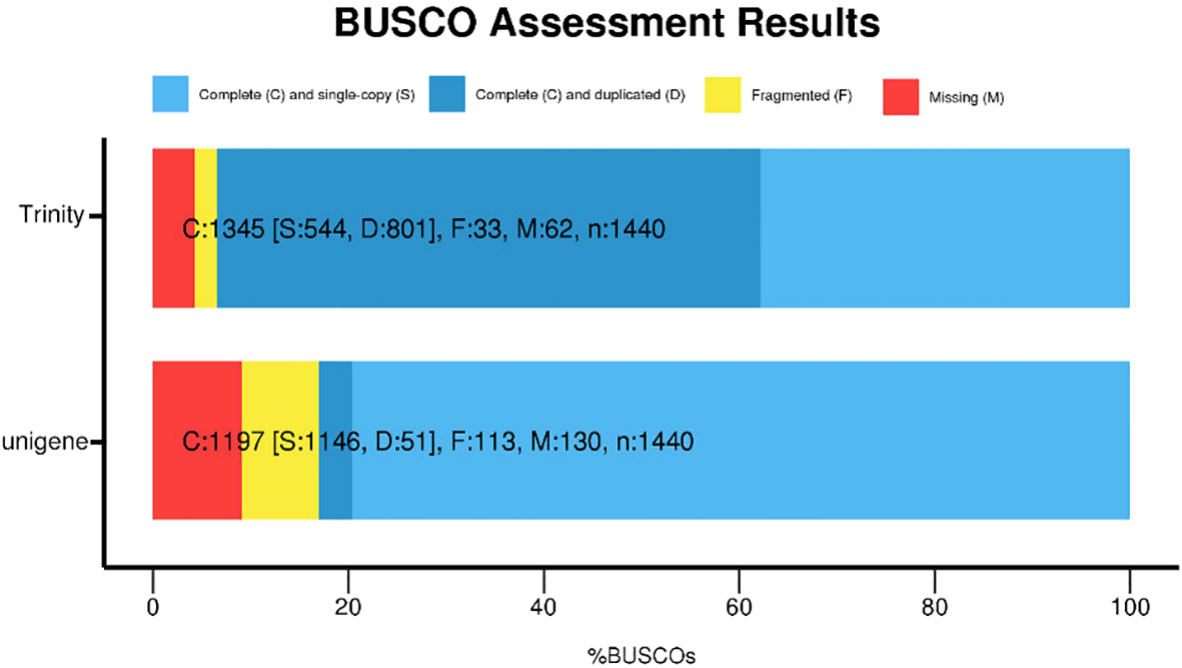
Benchmarking universal single-copy orthologs (BUSCO) evaluation of the *C. giganteum* transcriptome assembly.

Following the acquisition of high-quality clean reads, *de novo* transcriptome assembly was performed using Trinity (v2.14.0), generating 202,773 unigenes with an N50 of 982 bp. The assembled unigenes exhibited a maximum length of 36,573 bp and an average length of 805 bp (**Figure 2**), with 43,420 (21.4%) unigenes exceeding 1,000 bp and 12,414 (6.1%) surpassing 2,000 bp in length. Assembly completeness was rigorously evaluated via BUSCO (Benchmarking Universal Single-Copy Orthologs; embryophyta_odb10 database). The results demonstrated 79.6% complete single-copy orthologs, with only 9.1% (**Figure 3**) missing homologs, confirming high assembly integrity. This robust transcriptomic resource provides a reliable foundation for downstream gene expression profiling and functional annotation analyses.

The assembled transcripts were functionally annotated through BLASTx homology searches against seven major databases: NCBI non-redundant protein (Nr), nucleotide (Nt), Pfam, KOG/COG, Swiss-Prot, KEGG, and Gene Ontology (GO). Annotation results revealed 49,414 unigenes matched to the Nr database, 71,259 to Swiss-Prot, and 76,726 to GO terms. Additionally, 40,182 unigenes showed significant homology to KOG/COG entries. Collectively, 108,971 unigenes (53.74% of total assembled sequences) received annotations in at least one database, with cross-database annotation statistics detailed in **Table 1**.

**Table 1:** Annotation results of unigenes in the transcriptome of *C. giganteum*.

Database	Number of annotated Unigenes	Ppercentage of annotated Unigenes(%)
NR	49414	24.36
NT	56508	27.86
KEGG	36486	17.99
SwissProt	71259	35.14
PFAM	76726	37.83
GO	76726	37.83
KOG	40182	19.81
All Databases	10047	4.95
At least one Database	108971	53.74
Total Unigenes	202773	100

**Figure 4:**
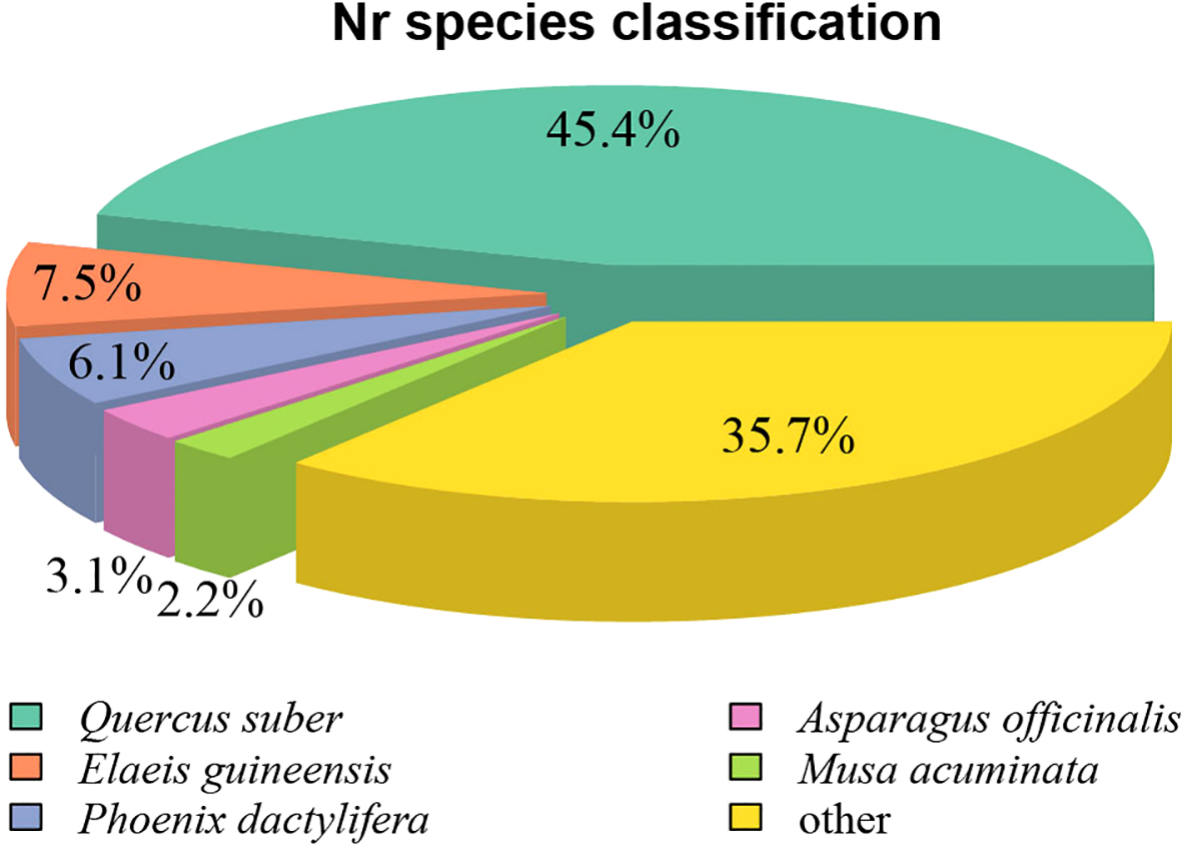
Species distribution map of the Nr annotation of the transcriptome of *C. giganteum*.

Homology analysis of the 49,414 Nr-annotated unigenes revealed significant sequence similarity to distantly related species (**Figure 4**). The highest homology was observed with *Quercus suber* (European cork oak; 45.4%, 40,569 sequences), followed by *Elaeis guineensis* (oil palm; 7.5%, 6,705 sequences) and *Phoenix dactylifera* (date palm; 6.1%, 5,478 sequences). This phylogenetic discrepancy likely stems from the scarcity of genomic data from closely related species within the *C. giganteum* family in public databases. The absence of high-quality reference genomes from taxonomically proximate species forced cross-family homology matching, directly contributing to the suboptimal annotation rate (53.74%) observed in this transcriptome study.

**Figure 5:**
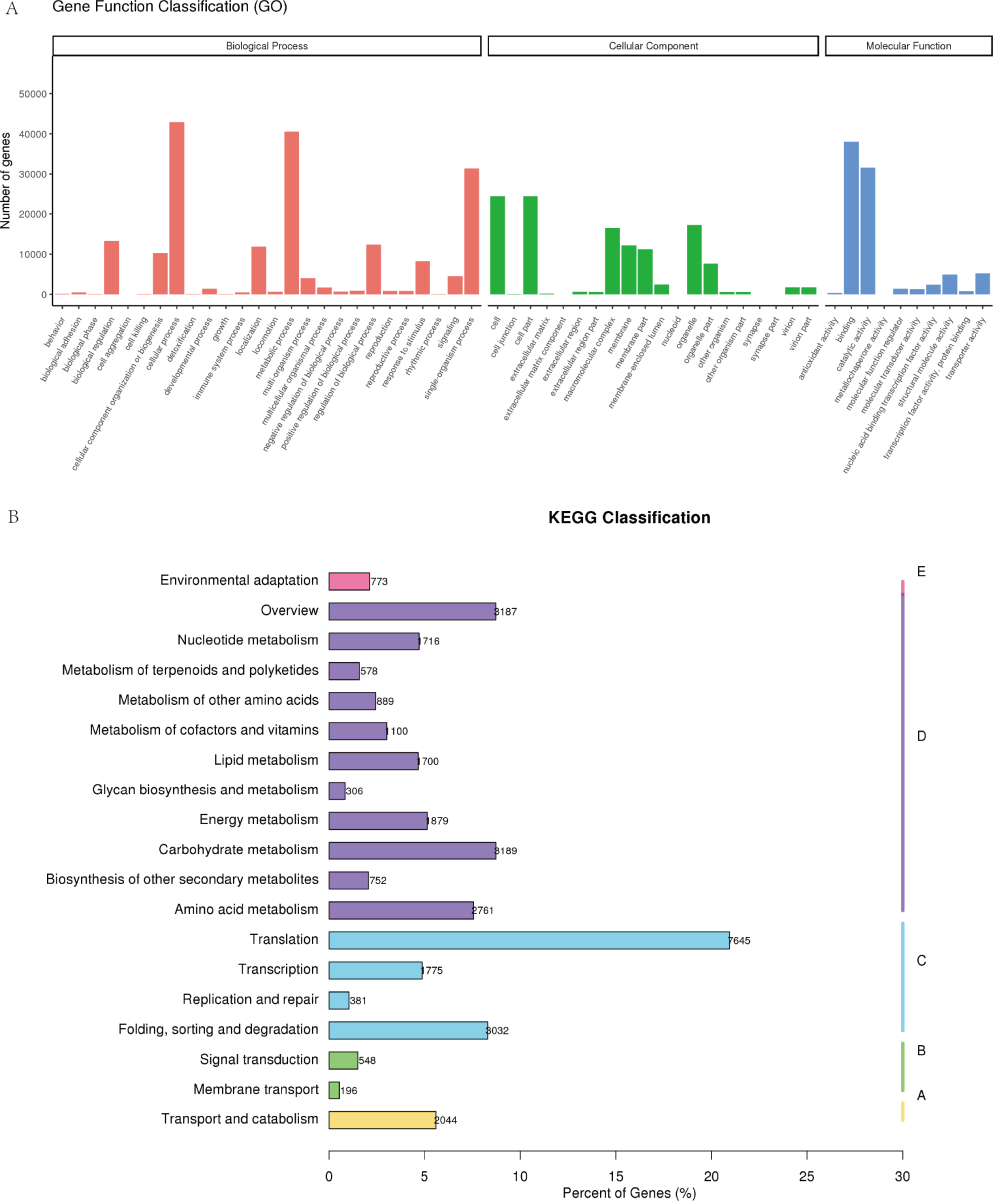
Classification of GO annotation and KEGG metabolic pathway annotation of unigenes genes in the transcriptome of *C*. *giganteum*. **(A)** GO annotation of unigenes in the transcriptome of *C*. *giganteum*; **(B)** KEGG metabolic pathway annotation of unigenes genes in the transcriptome of *C*. *giganteum*.

Based on Gene Ontology (GO) annotation, the transcripts were categorized into 46 functional subclasses spanning three primary GO categories: biological process, cellular component, and molecular function (**Figure 5A**). KEGG pathway enrichment analysis identified 36,486 genes annotated to 132 distinct metabolic pathways. Key pathways associated with *C. giganteum* seed germination and development included: Ribosome (4,906 genes), Carbon metabolism (1,887), Protein processing in endoplasmic reticulum (1,407), Plant-pathogen interaction (669), Glycolysis (702), Fatty acid metabolism (600), Starch and sucrose metabolism (389), Glycerolipid metabolism (275), and ABC transporters (196).This comprehensive functional annotation provides a critical foundation for elucidating molecular mechanisms underlying seed vigor and metabolic regulation in *C. giganteum*. The curated transcriptome resource enables targeted investigation of candidate genes governing dormancy release and germination physiology.3.3 Differentially expressed genes (DEGs) during post-maturation embryonic development in *C. giganteum* (**Figure 5B**).

### 3.3 Differentially expressed genes during post-maturation embryonic development in *C. giganteum*

**Figure 6:**
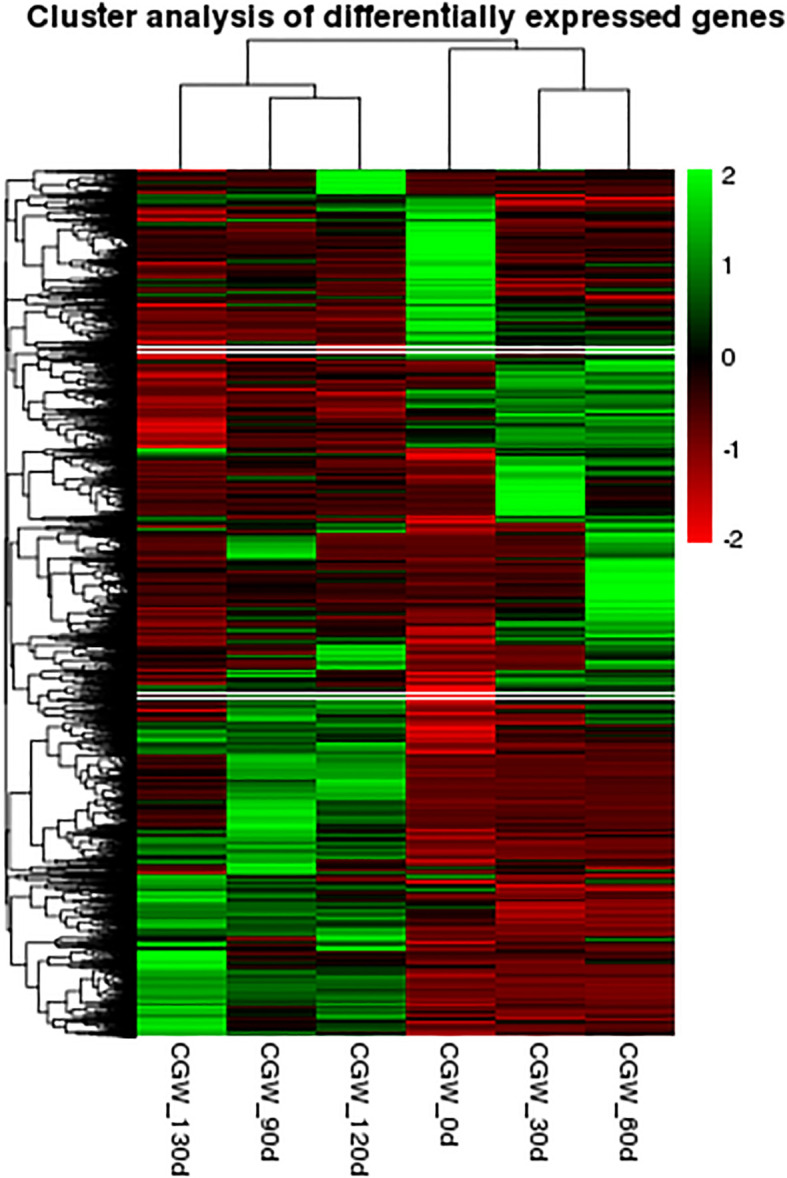
Heatmap analysis of globulin gene expression during the process of variable temperature stratification.

**Figure 7:**
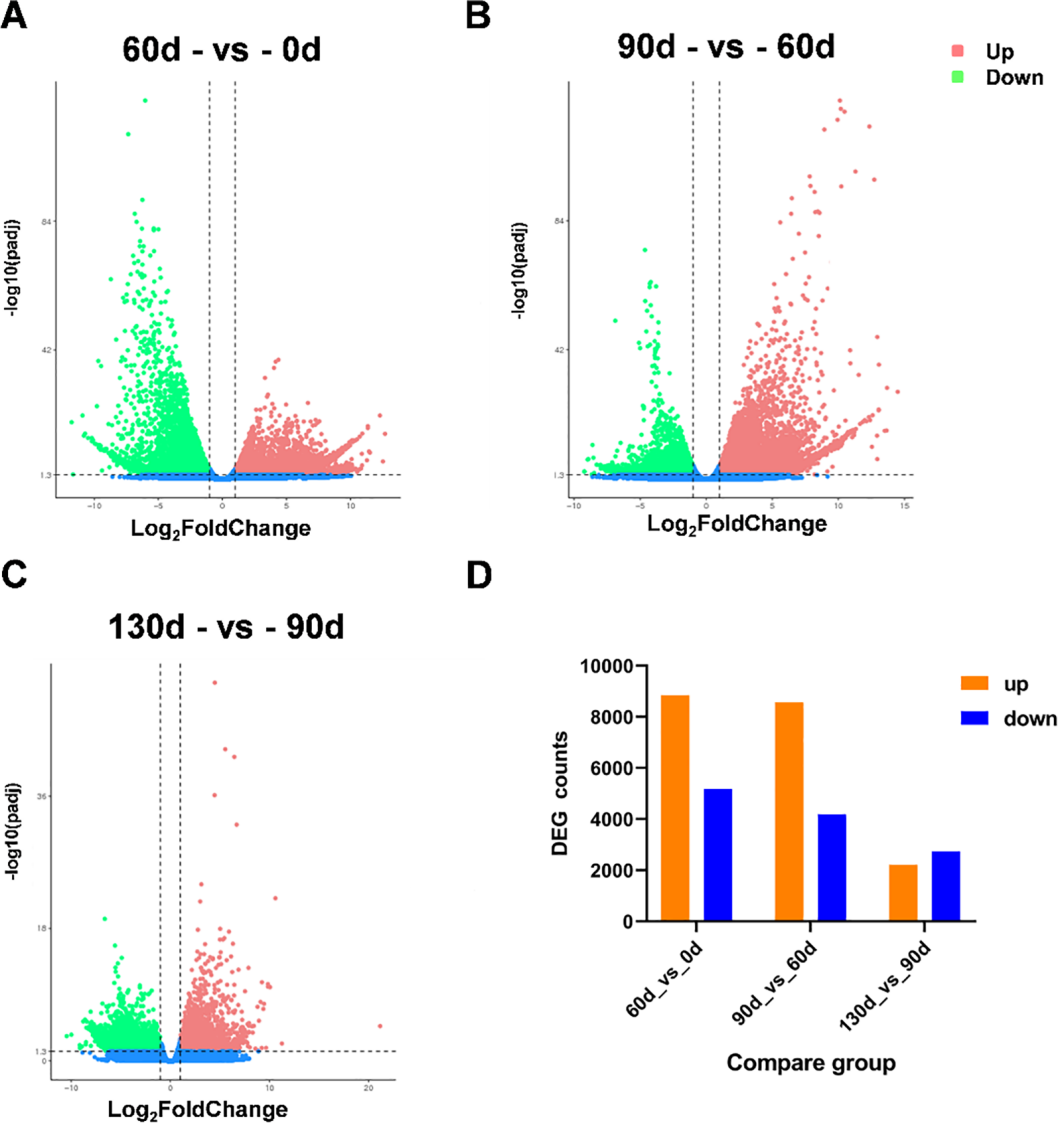
Differential gene expression profiles derived from pairwise comparisons of stratification time points. **(A–C)** the volcano plots of differential gene expression in the three comparison phases; **(D)** indicates the number of significantly differentially expressed genes in the three phases.

In this experiment, six distinct time points for the post-maturation development of the embryo were established, namely 0 d, 30 d, 60 d, 90 d, 120 d, and 130 d following the stratification of *C. giganteum* seeds for investigation. However, as indicated by all the gene expression heat maps assembled in this study (**Figure 6**), the gene expression profiles during the alternating temperature stratification at 30 d and 60 d were similar, and those at 120 d and 130 d were also comparable. To further elucidate the significant alterations of *C. giganteum* seeds in different germination processes, this study selected 0 d, 60 d, 90 d, and 130 d for further analysis. The expression quantities of *C. giganteum* seed samples at the four stages of alternating temperature stratification (0 d, 60 d, 90 d, and 130 d) were compared (**Figures 7A–C**). Using a significance level of P<0.05 and a fold change of |log2FoldChange|>1 as the screening criteria, the numbers of differentially expressed genes between each sample were obtained. As depicted in **Figure 7**, the numbers of up-regulated and down-Regulated genes were 8,838 up and 5,177 down at 60d vs 0d, 8,560 up and 4,179 down at 90d vs 60d, and 2,212 up and 2,736 down at 130d vs 90d (**Figure 7D**), indicating that a considerable number of genes were significantly differentially expressed in the early stage of *C. giganteum* seed germination.

**Figure 8:**
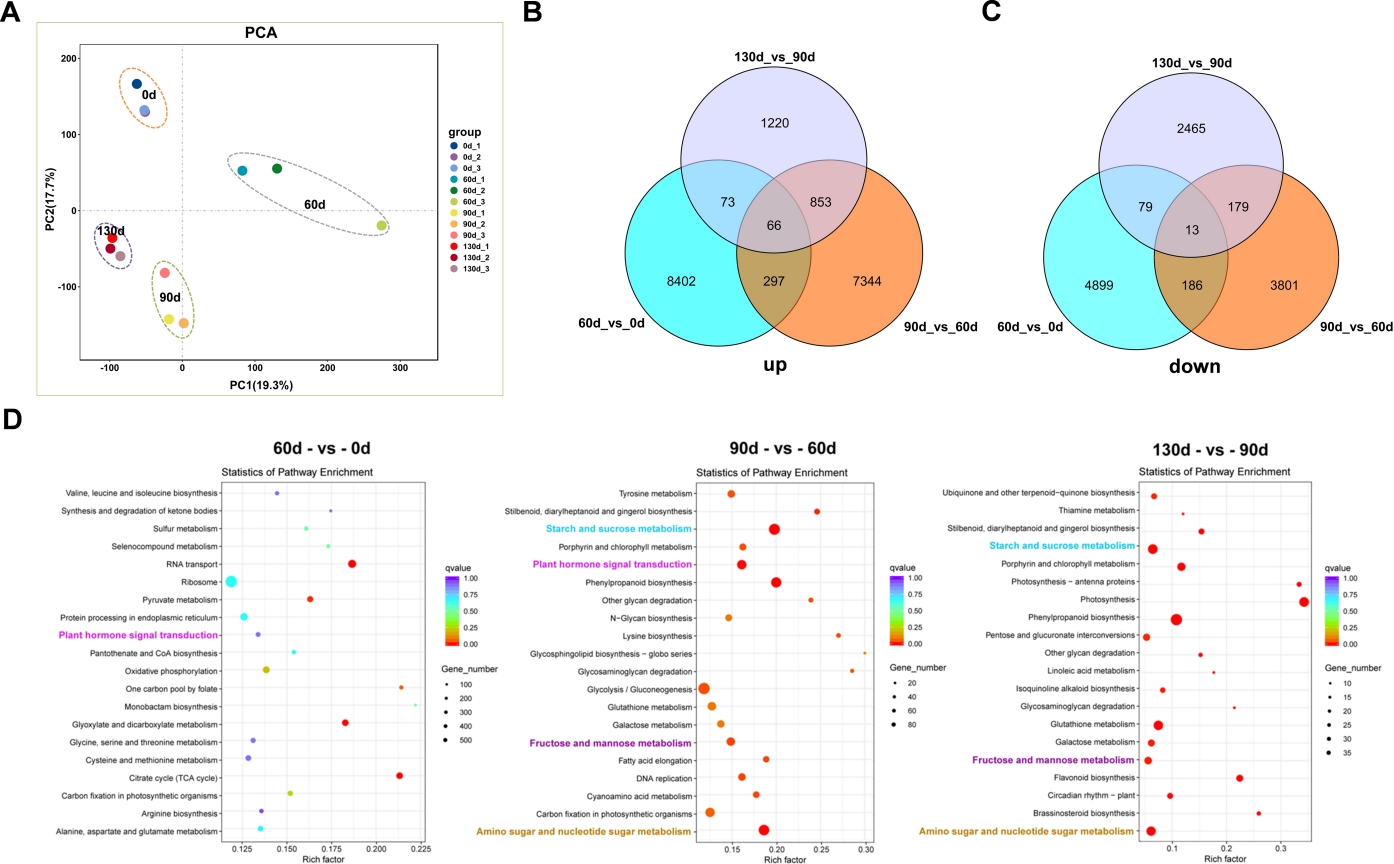
Analysis of differentially expressed genes during the seed germination process of *C. giganteum*. **(A)** PCA of 12 RNA-Seq samples; **(B)** Venn diagram showing the overlapping up-regulated DEGs in three comparison groups; **(C)** Venn diagram showing the overlapping down-regulated DEGs in three comparison groups; **(D)** the KEGG enrichment analysis diagram of the up-Regulated genes from the comparisons of the samples in the four periods.

Principal Component Analysis (PCA) of the 12 samples across four time points (**Figure 8A**) revealed high correlations among the three biological replicates within each time point, with replicate samples exhibiting strong similarity in expression patterns. Samples from the four distinct time points demonstrated significant differential expression, displaying divergent expression profiles. Gene expression patterns were tightly associated with seed stratification time; for example, gene expression profiles at 90 d and 130 d of alternating temperature stratification showed close similarity. Dynamic comparative analysis and Venn diagram intersection of significantly up- and down-regulated genes identified 66 upregulated genes and 13 downregulated genes (**Figures 8B, C**). Among the 66 upregulated genes, many were enriched in pathways related to development, hormone signal transduction, and carbohydrate metabolism, including Epidermis-specific secreted glycoprotein EP1, Aquaporin, Gibberellin-regulated protein 6, and Granule-bound starch synthase 1 (*GBSS1*). These genes exhibited sustained upregulation during the stratification-to-germination process in *C. giganteum* seeds (**Supplementary Table S1**).

Through conducting KEGG pathway enrichment analysis on the differentially expressed genes, we discovered that as the stratification process of *C. giganteum* seeds persisted, multiple pathways were significantly enriched. For example, Plant hormone signal transduction, Starch and sucrose metabolism, Fructose and mannose metabolism, and Amino sugar and nucleotide sugar metabolism were significantly differentially expressed in the later stages of seed embryo development (90 d, 120 d, 130 d) (**Figure 8D**). Meanwhile, Phenylpropanoid biosynthesis also exhibited a significant upward trend. Nevertheless, at the initial stage of seed stratification treatment, Plant hormone signal transduction, Starch and sucrose metabolism, and Amino sugar and nucleotide sugar metabolism were significantly down-regulated at 60 d.

### 3.4 Analysis of gene expression trends during seed germination in *C. giganteum*

**Figure 9:**
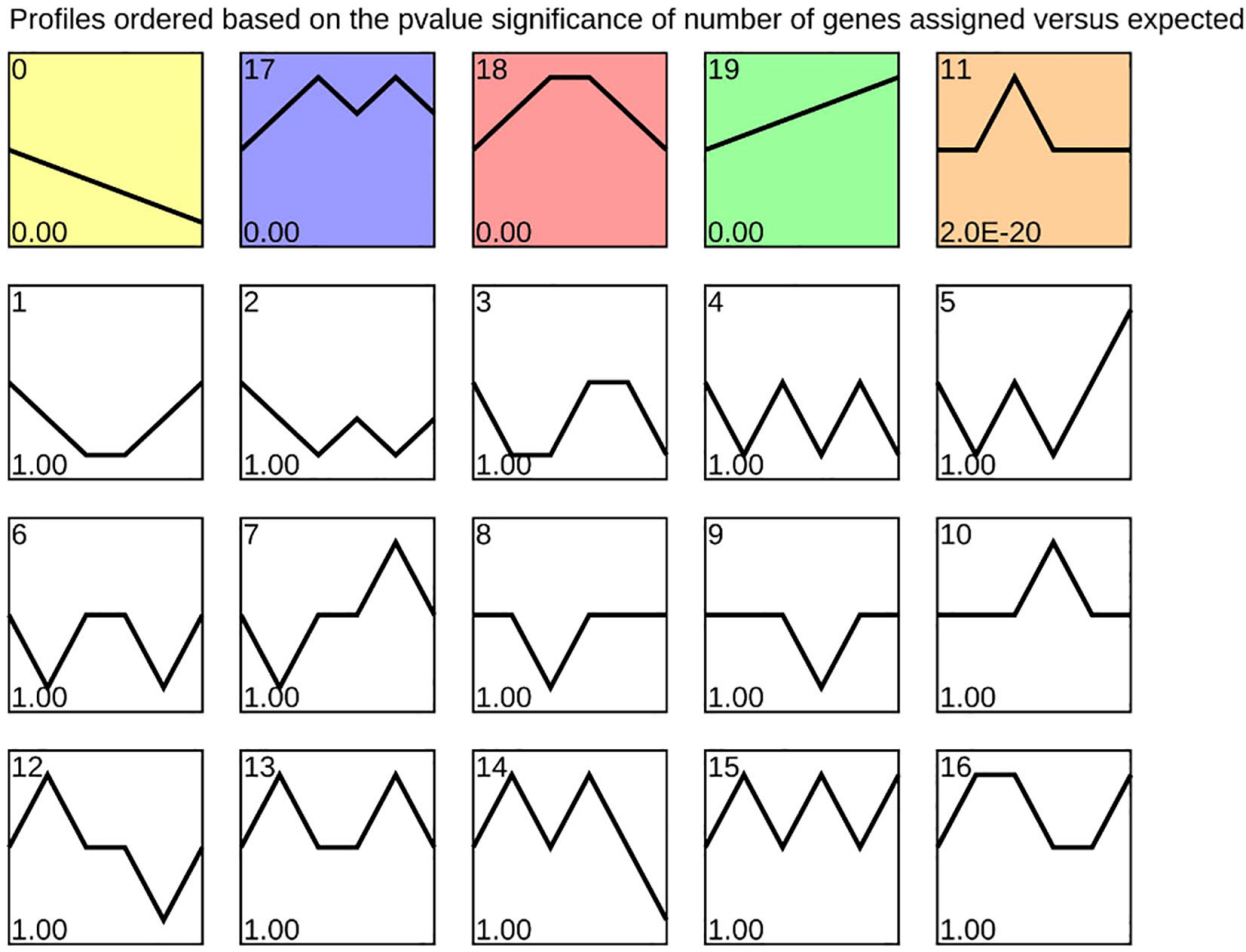
Hierarchical clustering analysis of temporally ordered expression patterns during alternating temperature stratification.

As stratification progressed during the alternating temperature stratification of *C. giganteum* seeds, seed development was initiated. To investigate temporal sequential expression patterns of differentially expressed genes (DEGs), we conducted expression trend analysis using STEM software on samples collected at six time phases (0 d, 30 d, 60 d, 90 d, 120 d, and 130 d). Clustering analysis revealed that the DEGs were partitioned into 20 distinct profiles (**Figure 9**), delineating global expression dynamics across the stratification treatment timeline. Notably, Profile 19 encompassed 32,143 genes exhibiting continuous upregulation at all six time points. Among these, lipid-transfer protein-encoding genes (Cluster-62345.49884 and Cluster-62345.49886) displayed 1,869-fold and 8,216-fold upregulation, respectively, from 0 d to 90 d. These findings suggest that stored lipids in the seeds undergo progressive transport and degradation to supply energy for germination during this process

Furthermore, Profile 0 encompassed 18,581 genes exhibiting a sustained downregulation pattern, most notably the globulin gene family, which encodes major seed storage proteins. Among these, 30 genes showed progressive downregulation throughout the stratification period, particularly between 120 d and 130 d. As shown in **Figure 6**, Cluster-62345.34414 and Cluster-62345.34338 were downregulated by 3.34-fold and 4.14-fold, respectively, at 120 days post-germination (120 d). Following subsequent low-temperature induction at 130 d, their expression decreased more markedly to 11.6-fold and 22.9-fold downregulation. These results demonstrate that low-temperature treatment beyond 120 d significantly suppressed globulin gene expression, thereby feedback-regulating and promoting seed germination.

### 3.5 Significantly differentially expressed genes associated with phytohormone signal transduction pathways during seed germination in *C. giganteum*

**Figure 10:**
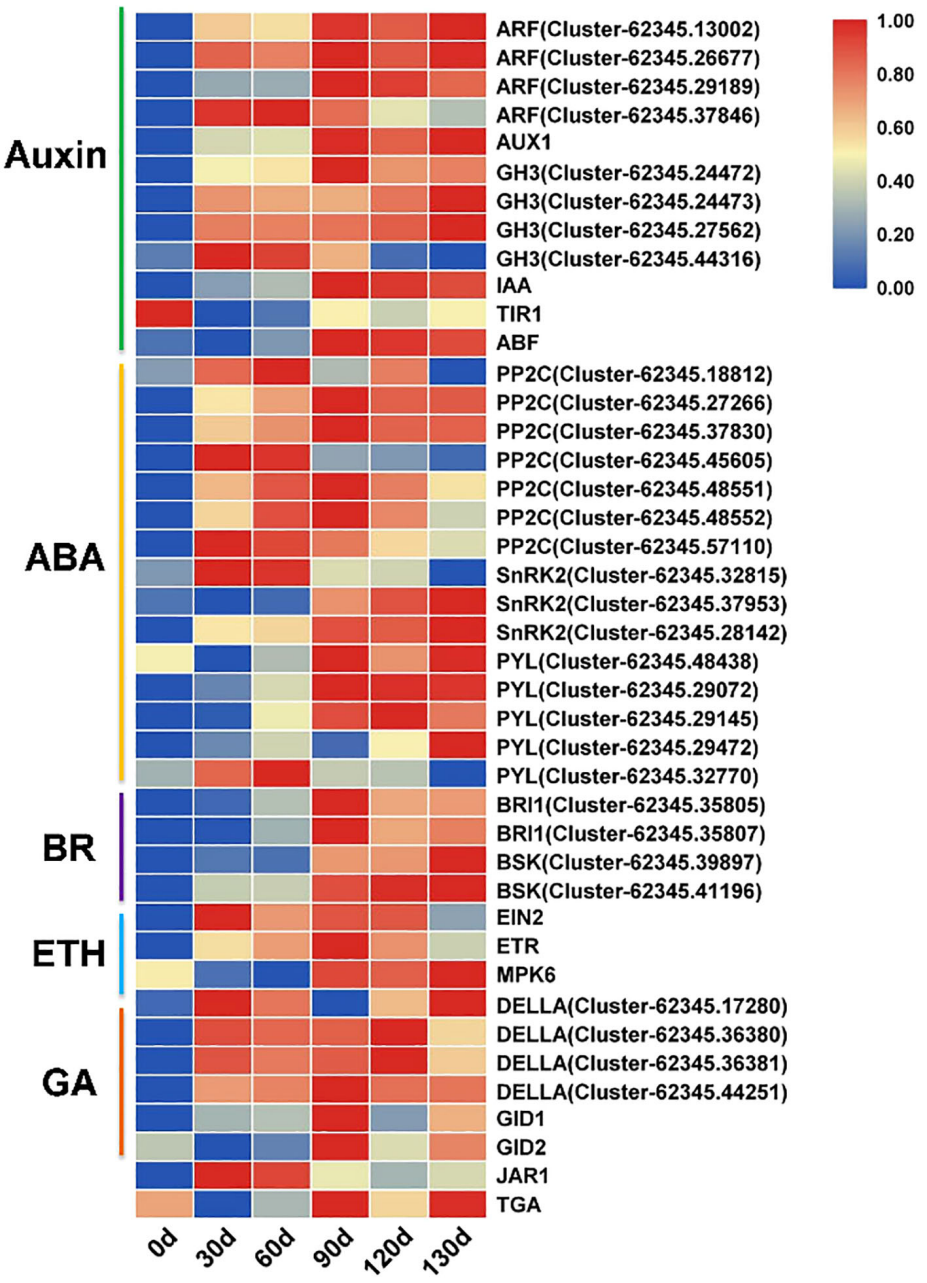
Expression profiles of key phytohormone signaling pathway genes during seed germination in *C. giganteum*.

Phytohormones play pivotal roles in seed germination. In this study, we identified 96 significantly differentially expressed genes (SDEGs) associated with hormone signaling transduction pathways. Among these, 46 genes were significantly expressed (FPKM≥1) and functionally linked to key components of auxin, abscisic acid (ABA), brassinosteroid (BR), ethylene, and gibberellin (GA) signaling pathways. At 0 d of stratification treatment, these genes showed no significant expression. From 30 d to 60 d, multiple genes exhibited initial upregulation, while pronounced differential expression emerged across these genes from 90 d to 130 d. For instance, in the auxin signaling pathway, *ARF* (Auxin Response Factor), *GH3*, and *IAA* genes were markedly upregulated after 90 d of stratification. Within the ABA signaling pathway, *PP2C* displayed an upward expression trend at 30 d, 60 d, and 90 d, whereas *SnRK2* and *PYL* exhibited sustained upregulation from 90 d to 130 d (**Figure 10**). Furthermore, *DELLA*-encoding genes in the GA signaling pathway demonstrated a progressive upregulation pattern during the stratification process of Cardiocrinum giganteum seeds.

### 3.6 Carbohydrate metabolism during seed germination in *C. giganteum*

**Figure 11:**
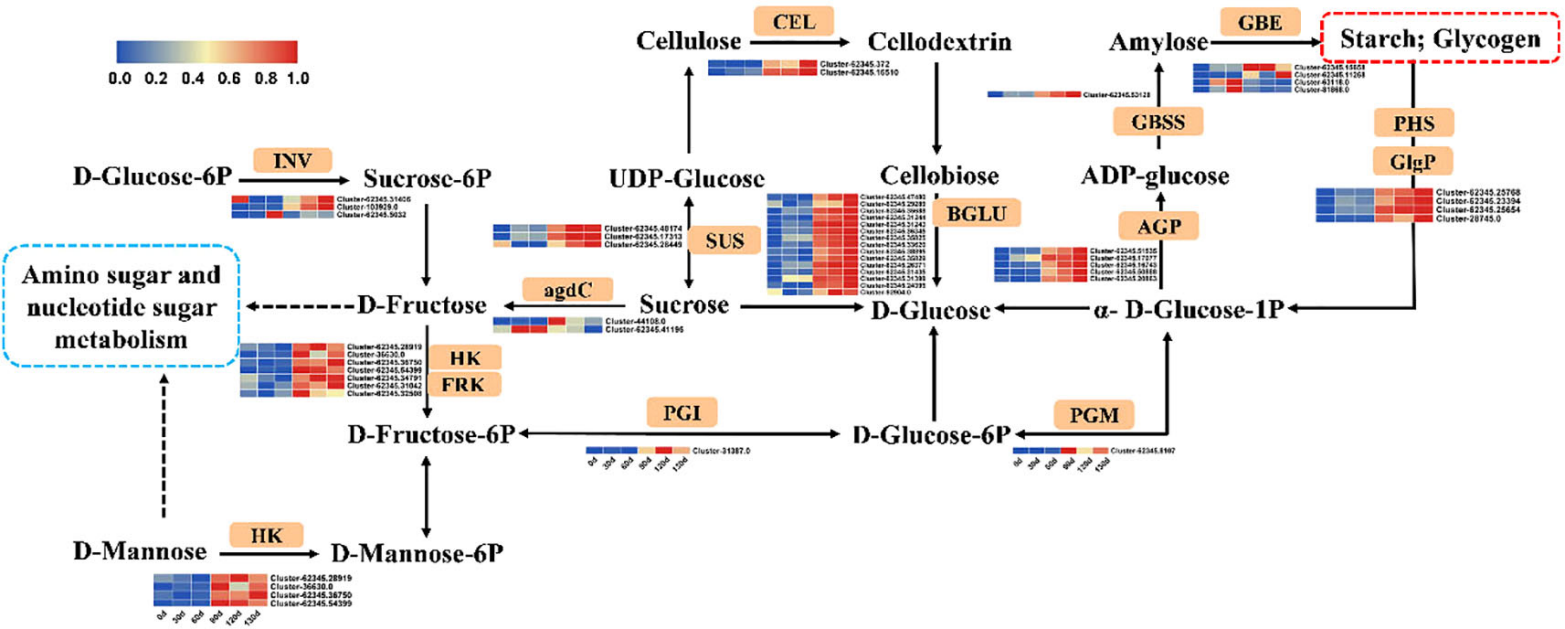
Schematic representation of carbohydrate metabolism and associated gene expression profiles during seed germination in *C. giganteum*.

KEGG enrichment analysis revealed significant enrichment of starch and sucrose metabolism, fructose and mannose metabolism, and amino sugar and nucleotide sugar metabolism pathways in *C. giganteum* seeds at 90 d, 120 d, and 130 d of stratification (**Figure 11**). Subsequent analysis of gene expression patterns across these three metabolic pathways demonstrated marked upregulation of multiple rate-limiting enzymes post-90d stratification. Notably, Beta-fructofuranosidase (*INV*), Hexokinase (*HK*), Fructokinase (*FRK*), Beta-glucosidase (*BGLU*), and Sucrose synthase (*SUS*) exhibited significant transcriptional activation during this phase.

In this study, Beta-glucosidase (*BGLU*) genes (Cluster-62345.33620, Cluster-62345.31435, and Cluster-62345.35688) associated with polyhydroxy hydrolysis exhibited progressive upregulation during stratification, reaching 6.68-, 5.08-, and 6.85-fold increases, respectively, at 130 d. Concurrently, activation of the glycolytic pathway was observed throughout the process, suggesting that *BGLU* enzymes likely catalyze the conversion of storage polysaccharides into glucose to fuel germination via glycolysis. Notably, stigma/stylar cysteine-rich adhesin genes (Cluster-62345.50062, Cluster-62345.49889, and Cluster-62345.49890), typically linked to pollen tube growth and adhesion, also showed marked upregulation. Specifically, Cluster-62345.49889 displayed a 1,464-fold increase at 90 d compared to 0 d, escalating to 5,573-fold upregulation after 130 d of low-temperature induction, implicating its potential regulatory role in germination. Furthermore, multiple gene families within the phenylpropanoid biosynthesis pathway—including *BGLU*, cinnamyl alcohol dehydrogenase (*CAD*), mannitol dehydrogenase, and peroxidase (*PER*)—exhibited significant differential expression. These findings collectively indicate that *BGLU* enzymes not only participate in phenylpropanoid biosynthesis but also play a critical role in carbohydrate metabolism (**Figure 11**).

### 3.7 The effect of the phenylpropanoid biosynthesis pathway on seed germination in *C. giganteum*

**Figure 12:**
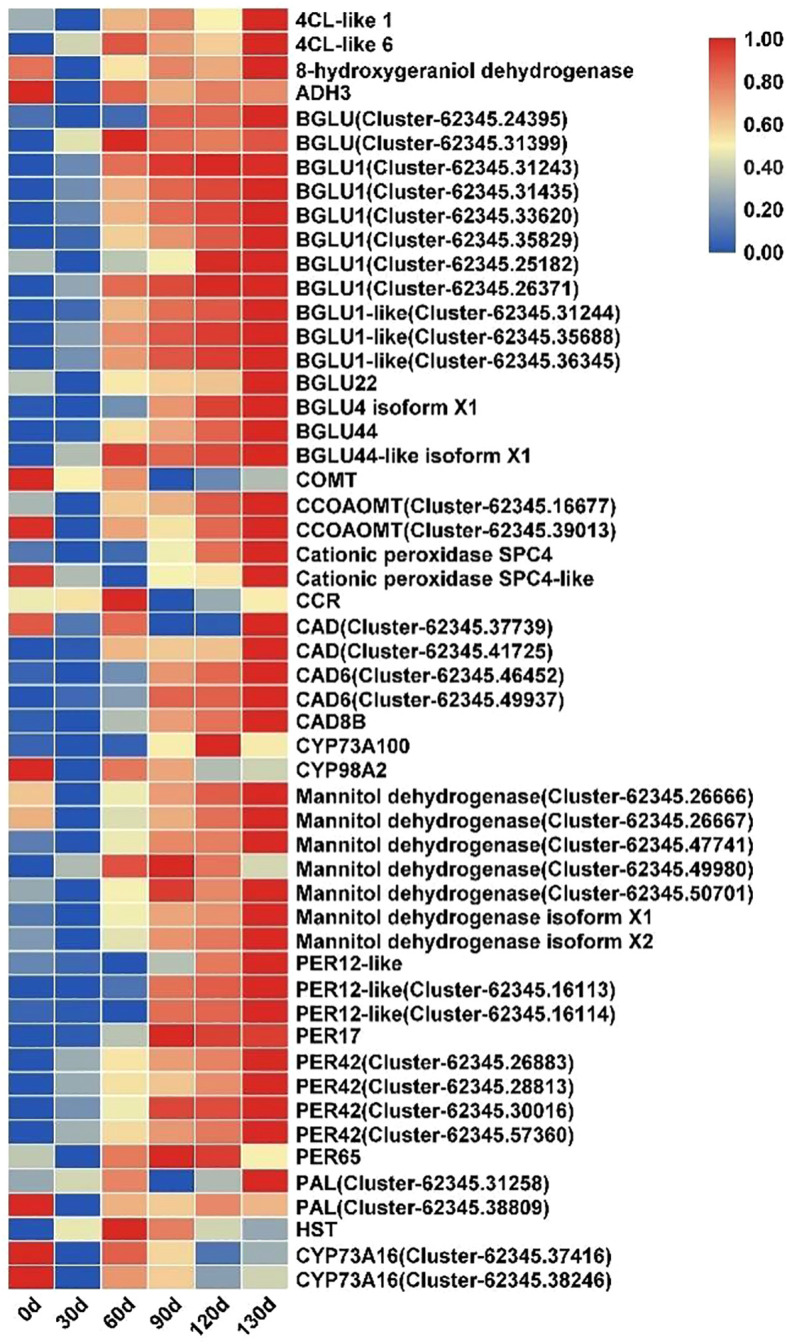
Significant differential expression of phenylpropanoid biosynthesis pathway genes during seed germination in *C. giganteum*.

The phenylpropanoid biosynthesis pathway plays a critical role in plant growth and development. In this study, multiple enzyme-encoding genes within this pathway exhibited sustained upregulation during the stratification of *C. giganteum* seeds. Key genes, including those encoding 4-coumarate-CoA ligase (*4CL*), Beta-glucosidase (*BGLU*), caffeoyl-CoA O-methyltransferase (*CCOAOMT*), cinnamyl alcohol dehydrogenase (*CAD*), mannitol dehydrogenase, and peroxidase (*PER*), demonstrated significant transcriptional activation starting at 60 d of stratification treatment (**Figure 12**). Their expression levels progressively increased throughout the treatment timeline, indicating that phenylpropanoid metabolism is actively involved in regulating seed germination in *C. giganteum*.

### 3.8 Verification of gene fluorescence quantification

**Figure 13:**
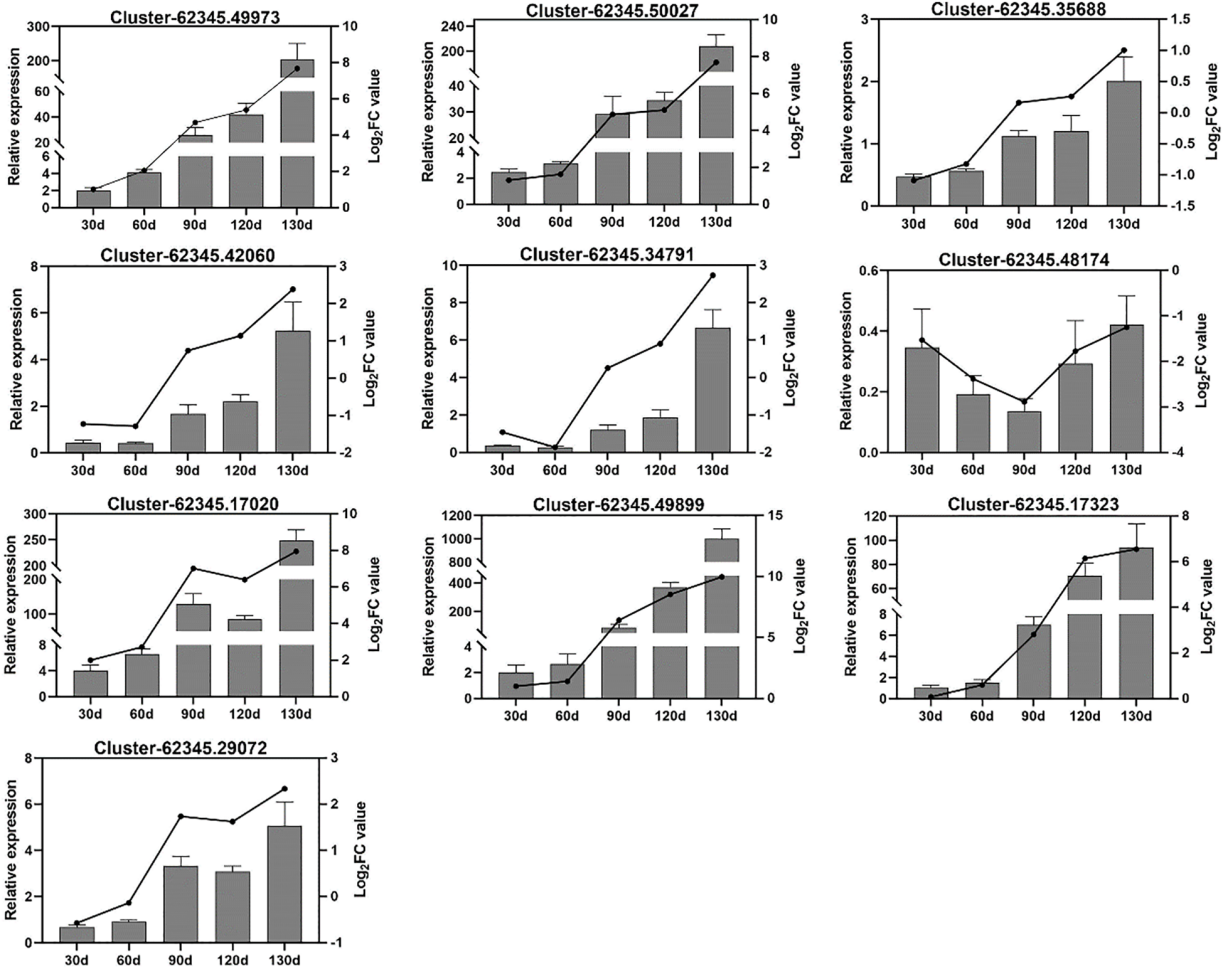
Validation of 10 candidate differentially expressed genes (DEGs) by qRT-PCR.

To further elucidate the gene expression dynamics during *C. giganteum* seed germination, we selected 10 significantly upregulated genes for validation via quantitative reverse transcription PCR (qRT-PCR). These genes encode enzymes critical to germination physiology: abscisic acid receptor PYL2-like, sucrose synthase A, beta-glucosidase 1-like, fructokinase-2, peroxidase 63 precursor, plasma membrane intrinsic protein, gibberellin-regulated protein 6-like isoform X1, tonoplast intrinsic protein, granule-bound starch synthase, cytochrome P450 90B1, and aquaporin PIP2-6. qRT-PCR results corroborated transcriptomic data, showing pronounced upregulation of these genes at 90 d post-stratification under alternating temperatures (**Figure 13**). This confirms their functional significance in *C. giganteum* seed germination, with most genes implicated in starch/sucrose metabolism and hormone signaling transduction pathways.

## 4 Discussion

### 4.1 The effect of alternating temperature stratification on the seed germination of *C. giganteum*

Our experimental findings demonstrate that temperature plays a decisive role in breaking seed dormancy in *C. giganteum*. Under natural conditions, *C. giganteum* seeds require 17–18 months spanning autumn-winter-spring-summer-winter-spring seasons to complete dormancy release and germination (Guan et al., 2010). As proposed by Baskin and Baskin (2004), morphologically dormant seeds typically exhibit underdeveloped embryos that are either undifferentiated or partially differentiated (with distinguishable cotyledons and radicles), requiring continued embryonic growth and organ differentiation prior to germination. At dispersal, *C. giganteum* embryos measure (1.06 ± 0.07) mm in length (Li et al., 2020), lacking discernible differentiation of radicles, cotyledons, hypocotyls, or plumules. This indicates that embryonic development remains incomplete at seed maturity—the primary cause of dormancy—necessitating prolonged alternating temperature regimes for full embryogenesis. These characteristics confirm that *C. giganteum* seeds exhibit morphological dormancy (MD). During sequential stratification treatments [25/15°C(60 d)→15/15°C(60 d)→5°C], *C. giganteum* embryos gradually differentiated under warm (25/15°C) to moderate (15/15°C) temperatures but required subsequent low-temperature exposure (5°C) to complete physiological after-ripening. This multi-phase requirement establishes *C. giganteum* seed dormancy as morphophysiological dormancy (MPD), the most complex dormancy category requiring both morphological and physiological maturation.

### 4.2 The regulatory role of plant hormones on the seed germination of *C. giganteum*

Phytohormones serve as critical regulators of seed germination, with endogenous plant hormones such as auxin, gibberellins (GA), and abscisic acid (ABA) being intimately associated with this process. The transition from seed dormancy to germination competence is governed by the dynamic equilibrium between ABA and GA levels. A reduced ABA/GA ratio facilitates dormancy release and promotes germination, with these hormones exerting antagonistic effects to modulate the germination process (Bewley et al., 2013). Emerging evidence suggests that an ABA-repressor complex may coordinate the suppression of ABA signaling to initiate germination in seeds (Nonogaki, 2020). Abscisic acid (ABA) maintains seed dormancy through a gene regulatory network mediated by ABA-insensitive (*ABI*) transcription factors. Conversely, gibberellins (GA) promote germination via GA-signaling pathway components such as *SLY1* and *GID1*. Environmental stressors—including drought and elevated temperatures—induce ABA biosynthesis to reinforce dormancy. Under low-temperature conditions, DOG1-mediated signaling enhances *ABI3* expression while negatively regulating GA biosynthesis. Furthermore, auxin signaling modulates ABA homeostasis through *ARFs* (Auxin Response Factors) under the post-transcriptional control of miR160, thereby upregulating ABA biosynthetic and signaling genes (Reed et al., 2022). In *Arabidopsis* studies, the core mechanism of seed dormancy is mediated by ABA signaling through phosphorylation-dependent activation of kinases, *SnRK2* family members, and transcription factors such as *ABI5* (Umezawa et al., 2004, 2009; Nakashima et al., 2009). DELAY OF GERMINATION 1 (*DOG1*), a central regulator of seed dormancy, suppresses germination by inactivating *AHG1-PP2C* phosphatase activity (Née et al., 2017; Nishimura et al., 2018; Nonogaki, 2020).

In this study, genes encoding *ARFs*, *AUX1*, *GH3*, and *IAA* family components within the auxin signaling transduction pathway exhibited progressive upregulation with prolonged stratification duration. Similarly, *BRI1* and *BSK* in the brassinosteroid (*BR*) signaling pathway showed gradual transcriptional activation as stratification time increased.

### 4.3 Sugar metabolism plays a pivotal role in the seed germination of *C. giganteum*

The carbohydrate metabolism involving the internal stored substances of seeds plays a crucial role in seed germination. For example, through the delay of starch and other metabolite degradation at low temperatures, it influences glycolysis and several other metabolic processes, thereby suppressing the germination of ‘Fengdan’ seeds of Paeonia lactiflora (Ren et al., 2018). In *Castanea henryi* seeds, granule-bound starch synthase (*GBSS*) and sucrose synthase (*SUSY*) serve as key regulators of sucrose-starch interconversion, with *GBSS* likely governing starch biosynthesis. β-amylase (*BAM*), a critical starch-degrading enzyme, and *SUSY*, which catalyzes sucrose cleavage into UDP-glucose and fructose during germination, coordinately drive carbohydrate mobilization to fuel embryonic growth (Winter et al., 1997; Zhao et al., 2018; Liu et al., 2020).

Our findings revealed that prolonged alternating-temperature stratification of *C. giganteum* seeds induced upregulation of numerous carbohydrate metabolism-associated genes, triggering the synthesis or activation of critical hydrolases. These enzymes facilitate energy mobilization through enzymatic hydrolysis of storage reserves to fuel germination (Zhao et al., 2021). Notably, β-glucosidase gene expression increased significantly, catalyzing the conversion of macromolecules (starch, sucrose) into glucose. Sucrose metabolism was governed by two key enzymes: sucrose-phosphate synthase (*SPS*) and sucrose synthase (*SUSY*), which respectively regulate sucrose biosynthesis and catabolism. In *Idesia polycarpa* seeds, substantial upregulation of *SPS* and *SUSY* genes during later germination stages drove massive sucrose synthesis, followed by its enzymatic cleavage into glucose and fructose via SUSY-mediated catalysis (Zhang et al., 2024). The expression levels of key enzyme genes involved in starch and sucrose metabolism were strongly correlated with dynamic changes in starch and soluble sugar contents within seeds. In *Castanea henryi* seeds, most genes associated with starch and soluble sugar biosynthesis exhibited decreasing expression trends, while those linked to degradation pathways showed increasing expression patterns (Liu et al., 2020). Imbibition of *Zea* mays seeds in the presence of the α-galactosidase inhibitor 1-deoxygalactonojirimycin resulted in significantly elevated raffinose and galactitol accumulation compared to aqueous controls, correlating with reduced germination rates (Zhang et al., 2021). Raffinose family oligosaccharides (*RFOs*), which accumulate during late seed development and undergo hydrolysis during germination, are proposed to maintain sucrose homeostasis while contributing to reducing sugar pools that energize germination (Gu et al., 2019). Differential expression of carbohydrate metabolism-related genes (DEGs) critically regulates germination initiation and establishment, though their precise regulatory networks remain to be fully elucidated.

### 4.4 Phenylpropanoid biosynthesis pathway genes play a critical regulatory role in the seed germination of *C. giganteum*

It is widely recognized that phenylpropanoid biosynthesis exerts a positive regulatory effect on seed germination through multifaceted mechanisms (Yan et al., 2020). The phenylpropanoid pathway, a complex secondary metabolic route involving multiple gene families. This study analyzed differentially expressed genes (DEGs) associated with the phenylpropanoid biosynthesis pathway to delineate their functional roles in dormancy release of *Cardiocrinum giganteum* seeds. KEGG pathway analysis revealed significant enrichment of multiple DEGs in phenylpropanoid biosynthesis, suggesting this pathway may function pivotally in alternating-temperature stratification-induced dormancy alleviation. Wang et al. (2024) reported congruent findings, demonstrating that phenylpropanoid biosynthesis plays a critical role in seed germination of *Suaeda glauca*. During dormancy release, most differentially expressed genes (DEGs) associated with this pathway—including *SgPER3*, *SgPER4*, *SgPER12*, *SgCNMT*, and *SgBGH3B17*—exhibited significant upregulation. Tong et al. (2021) demonstrated that bruceine D inhibits *Bidens pilosa* L. seed germination by suppressing key enzymatic activities in the phenylpropanoid biosynthesis pathway. Notably, phenylalanine ammonia-lyase (*PAL*), cinnamate 4-hydroxylase (*C4H*), and 4-coumarate-CoA ligase (*4CL*) genes exhibited temporal expression fluctuations, while caffeic acid O-methyltransferase (*COMT*) showed significant downregulation during bruceine D treatment - a regulatory pattern consistent with our current observations. Peroxidases (*PERs*), core enzymatic components of the phenylpropanoid biosynthesis pathway, exhibited progressive upregulation (*PER12*, *PER17*, *PER42*) during dormancy alleviation in treated *C. giganteum* seeds. This regulatory pattern correlated with a concurrent decline in reactive oxygen species (ROS) levels and subsequent germination initiation, paralleling observations in low-temperature stress-delayed germination responses of *Zea* mays seeds (Meng et al., 2022). β-Glucosidases (*BGLUs*) mediate the regulatory shift from activation to suppression of phenylpropanoid biosynthesis-associated genes (Shao et al., 2022). Our investigation revealed substantial upregulation of *BGLU*-related differentially expressed genes (DEGs) during *C. giganteum* seed dormancy release, with concomitant involvement in carbohydrate metabolism. As reported by Sukanya Luang et al. (Luang et al., 2013), the rice (*Oryza sativa*) *Os9BGlu31*—a glycoside hydrolase family 1 (*GH1*) transglycosidase—catalyzes glucosyl transfer among phenolic acids, phytohormones, and flavonoids. We thus hypothesize that *BGLUs* may coordinate the metabolic equilibrium between phenylpropanoids, flavonoids, and carbohydrates. Elucidating the sophisticated regulatory network governing phenylpropanoid biosynthesis during *C. giganteum* dormancy alleviation will provide critical theoretical foundations for developing dormancy-breaking strategies.

### 4.5 The impact of lipid transfer proteins on seed germination

Numerous studies have indicated that lipid transfer proteins (LTPs) play a role in influencing the development and germination of plant seeds. In research on rice, upon knockout of the lipid transfer protein OsLTPL18, the seeds of the osltpl18 mutant exhibited a thinner phenotype. Specifically, the thousand - grain weight and grain thickness of the plants were significantly reduced, and the seed vigor of osltpl18 seeds declined notably (Li et al., 2023b). In the OsLTPL36 RNAi lines, seed germination was impeded, and the seedlings were frail. The lipid transfer protein OsLTPL36 is of vital significance for seed quality, seed development, and germination in rice (Wang et al., 2015). In rice, OsLTPL23 potentially influences rice seed germination and the early growth of seedlings by modulating endogenous ABA homeostasis (Li et al., 2023a). In *Arabidopsis thaliana*, LTPG15 participates in the export of suberin monomers in the seed coat, thereby influencing the permeability of the Arabidopsis seed coat (Lee and Suh, 2018). It remains to be further investigated how lipid transfer proteins affect the seed development and germination of *C. giganteum*.

### 4.6 Temperature serves as a pivotal environmental regulator governing seed germination in *C. giganteum*

Temperature serves as a critical environmental synchronizer of seed dormancy and germination, mediating plant developmental alignment with seasonal climatic shifts (Kendall and Penfield, 2012). Seed dormancy constitutes an adaptive strategy to suboptimal environments, manifesting as arrested germination under unfavorable thermal regimes. Viable seeds exposed to thermal extremes (e.g. supraoptimal temperatures) may undergo germination competency loss, thereby inducing secondary dormancy (Hilhorst et al., 2010; de Vries et al., 2021). As a primary environmental determinant of dormancy depth, temperature fluctuations as subtle as 1°C within thermosensitive thresholds can determine dormancy status (Springthorpe and Penfield, 2015). Seeds developing under extreme temperatures typically exhibit enhanced dormancy phenotypes (Toh et al., 2008; Penfield and MacGregor, 2016). In rice (*Oryza sativa*), high-temperature and drought stresses promote abscisic acid (ABA) accumulation through induced expression of 9-cis-epoxycarotenoid dioxygenases (*NCEDs*), consequently suppressing germination (Liu et al., 2019; Suriyasak et al., 2020), NCEDs serve as multifunctional regulators mediating seed dormancy, vegetative growth, abiotic stress tolerance, and leaf senescence (Huang et al., 2018).

### 4.7 Annotation of unigenes in the *C. giganteum* transcriptome

In this study, a total of 108,971 genes were annotated in at least one database, accounting for 53.74% of all assembled Unigenes. This finding suggests that nearly half of the unigenes remained unannotated, implying that their functions could not be resolved. The relatively low annotation rate might be attributed to the scarcity of gene libraries of species closely related to *C. giganteum* in existing databases. However, despite the fact that a substantial number of genes lack functional annotations, we did succeed in measuring the expression sequences and expression levels of these genes. This information is not without significance for deciphering their roles in dormancy. Instead, it indicates the potential existence of novel genes that are likely associated with dormancy, thereby necessitating further in - depth investigation. To enhance the gene annotation rate, we put forward the following recommendations: It is advisable to relax the criteria for gene alignment in order to identify potential homologous genes and utilize their functions as references.

## 5 Conclusions

**Figure 14:**
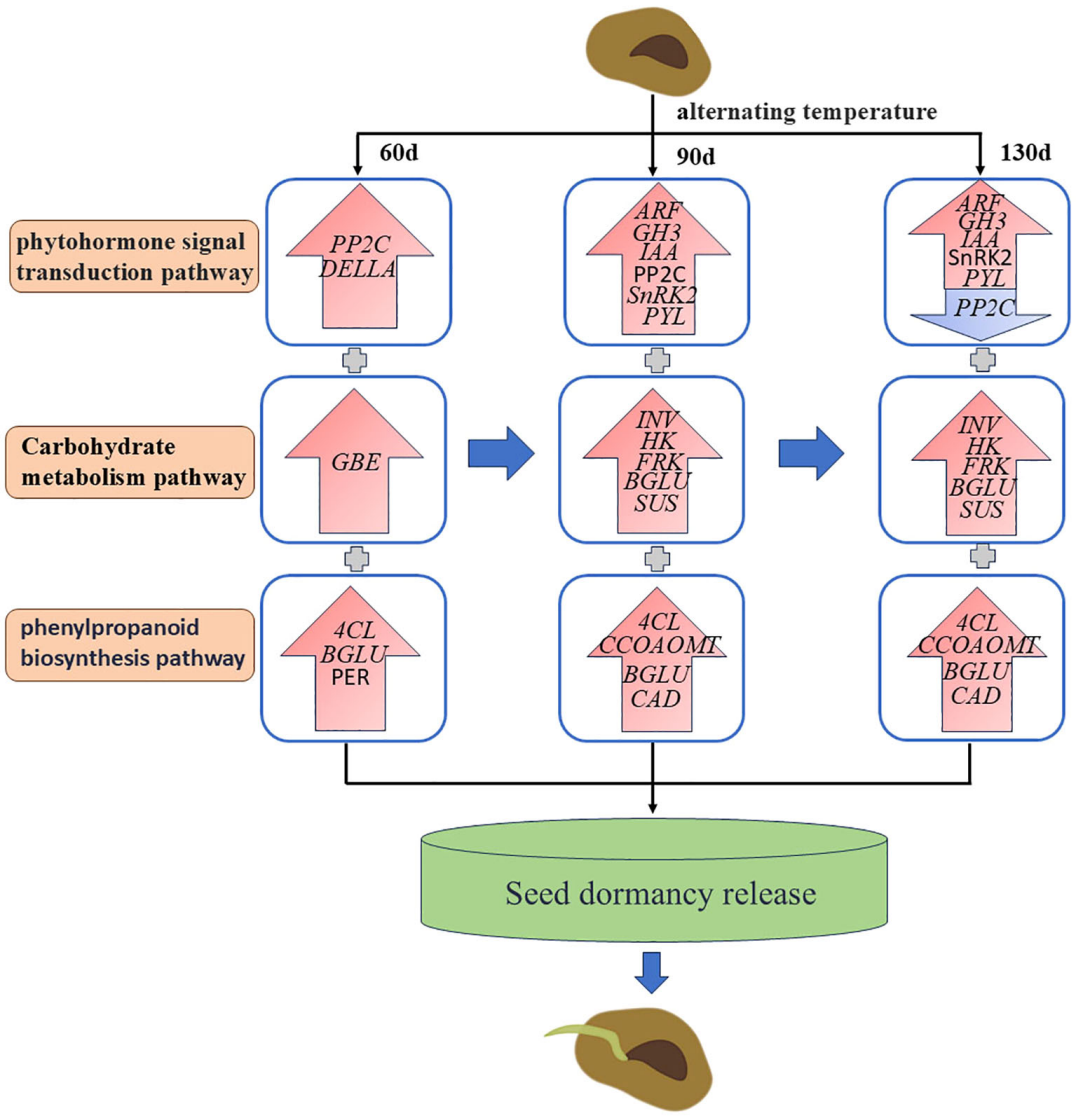
Regulation model of the dormancy release of *C. giganteum* seeds induced by alternating temperatures.

This study pioneers the application of transcriptomic approaches to investigate regulatory mechanisms underlying post-maturation developmental processes in *C. giganteum* embryos. We conducted comprehensive analysis of transcriptome profiles across four distinct embryonic development stages, proposing an alternating-temperature-induced dormancy release model (**Figure 14**). Following 30-day thermal cycling treatment, phytohormone signaling pathways—including auxin, abscisic acid (ABA), brassinosteroid (BR), ethylene, and gibberellin—exhibited coordinated activation through upregulated genes such as *PP2C* in ABA signaling. Core regulators (*ARF*, *GH3*, *IAA*, *SnRK2*, *PYL*, *DELLA*) orchestrated the thermoregulatory modulation of embryo maturation and germination competence. Following 90 days of alternating-temperature treatment, multiple genes including Epidermis-specific secreted glycoprotein *EP1*, Aquaporin, Gibberellin-regulated protein 6, GBSS1, Beta-fructofuranosidase (*INV*), Hexokinase (*HK*), Fructokinase (*FRK*), Beta-glucosidase (*BGLU*), and Sucrose synthase (*SUS*) were identified as critical regulators during *Cardiocrinum giganteum* seed germination. Aquaporin *PIP* and *TIP* isoforms synergistically modulated cellular osmotic regulation throughout seed maturation and germination, facilitating embryonic development (Mangrauthia et al., 2016; Zaghdoud et al., 2023; O’Lone et al., 2024). Concurrently, starch and sucrose metabolism pathway genes provided essential energy substrates for *Cardiocrinum giganteum* seed germination. At 120 days post-treatment, low-temperature induction upregulated key phenylpropanoid-related genes including 4-coumarate-CoA ligase (*4CL*), *BGLU*, caffeoyl-CoA O-methyltransferase (*CCOAOMT*), cinnamyl alcohol dehydrogenase (*CAD*), mannitol dehydrogenase, and peroxidase (*PER*), facilitating embryonic growth. By approximately 130 days of alternating-temperature treatment, seeds completed morphological and physiological post-maturation processes, with lipid-transfer protein (*LTP*) potentially mediating cellular membrane remodeling. This study advances our understanding of embryonic developmental regulation in *C. giganteum* while proposing innovative strategies for dormancy manipulation and breeding cycle acceleration.

## Data Availability

The data presented in the study are deposited in the NCBI repository, accession number PRJNA1285652. This data can be found here: https://dataview.ncbi.nlm.nih.gov/object/PRJNA1285652.

## References

[B1] Baskin C. C. Baskin J. M. (2014). Seeds Ecology, Biogeography, and Evolution of Dormancy and Germination Second Edition Introduction (London: Academic Press Ltd-Elsevier Science Ltd). doi: 10.1016/B978-0-12-416677-6.00001-9

[B2] Baskin J. M. Baskin C. C. (2004). A classification system for seed dormancy. Seed Sci. Res. 14, 1–16. doi: 10.1079/SSR2003150

[B3] Bewley J. D. Bradford K. J. Hilhorst H. W. M. Nonogaki H. (2013). Seeds: Physiology of Development, Germination and Dormancy. 3rd Edition (New York, NY: Springer New York). doi: 10.1007/978-1-4614-4693-4

[B4] Chen D. L. Luo X. P. Yuan Z. Bai M. J. Hu X. W. (2020). Seed dormancy release of *Halenia elliptica* in response to stratification temperature, duration and soil moisture content. BMC Plant Biol. 20, 352. doi: 10.1186/s12870-020-02560-8, PMID: 32723291 PMC7388213

[B5] de Vries S. Fuerst-Jansen J. M. R. Irisarri I. Ashok A. D. Ischebeck T. Feussner K.. (2021). The evolution of the phenylpropanoid pathway entailed pronounced radiations and divergences of enzyme families. Plant J. 107, 975–1002. doi: 10.1111/tpj.15387, PMID: 34165823

[B6] Gu L. Jiang T. Zhang C. Li X. Wang C. Zhang Y.. (2019). Maize HSFA2 and HSBP2 antagonistically modulate raffinose biosynthesis and heat tolerance in Arabidopsis. Plant J. 100, 128–142. doi: 10.1111/tpj.14434, PMID: 31180156

[B7] Guan W. Li S. Chen X. Li Y. Ke F. (2010). Dormancy characteristics and dormancy break of *cardiocrinum giganteum* seed. Acta Botanica Boreali-Occidentalia Sin. 30, 2479–2483.

[B8] Guan W. Li S. Li Y. Chen X. Li D. (2011). Analysis and assessment for nutrient contents in bulb of *cardiocrinum giganteum* . J. West China Forestry Sci. 40, 8–11. doi: 10.16473/j.cnki.xblykx1972.2011.01.005

[B9] Hilhorst H. W. M. Finch-Savage W. E. Buitink J. Bolingue W. Leubner-Metzger G. (2010). “Dormancy in plant seeds,” in Dormancy and Resistance in Harsh Environments. Eds. Lubzens E. Cerda J. Clark M. (Springer-Verlag Berlin, Berlin), 43–67. doi: 10.1007/978-3-642-12422-8_4

[B10] Huang Y. Guo Y. Liu Y. Zhang F. Wang Z. Wang H.. (2018). 9-cis-epoxycarotenoid dioxygenase 3 regulates plant growth and enhances multi-abiotic stress tolerance in rice. Front. Plant Sci. 9. doi: 10.3389/fpls.2018.00162, PMID: 29559982 PMC5845534

[B11] Kendall S. Penfield S. (2012). Maternal and zygotic temperature signalling in the control of seed dormancy and germination. Seed Sci. Res. 22, S23–S29. doi: 10.1017/S0960258511000390

[B12] Klupczyńska E. A. Pawłowski T. A. (2021). Regulation of seed dormancy and germination mechanisms in a changing environment. Int. J. Mol. Sci. 22, 1357. doi: 10.3390/ijms22031357, PMID: 33572974 PMC7866424

[B13] Kondo T. Sato C. Baskin J. M. Baskin C. C. (2006). Post-dispersal embryo development, germination phenology, and seed dormancy in *Cardiocrinum cordatum* var. *glehnii* (Liliaceae s. str.), a perennial herb of the broadleaved deciduous forest in Japan. Am. J. Bot. 93, 849–859. doi: 10.3732/ajb.93.6.849, PMID: 21642147

[B14] Lee S. B. Suh M. (2018). Disruption of glycosylphosphatidylinositol-anchored lipid transfer protein 15 affects seed coat permeability in Arabidopsis. Plant J. 96, 1206–1217. doi: 10.1111/tpj.14101, PMID: 30242928

[B15] Li Y. Guo L. Cui Y. Yan X. Ouyang J. Li S. (2023b). Lipid transfer protein, OsLTPL18, is essential for grain weight and seed germination in rice. Gene 883, 147671. doi: 10.1016/j.gene.2023.147671, PMID: 37506985

[B16] Li M. Ling K.-H. Lam H. Shaw P.-C. Cheng L. Techen N.. (2010). Cardiocrinum seeds as a replacement for Aristolochia fruits in treating cough. J. Ethnopharmacology 130, 429–432. doi: 10.1016/j.jep.2010.04.040, PMID: 20435131

[B17] Li Y.-F. Song J. Guan W.-L. Li F.-R. (2020). Seed dormancy and germination in *Cardiocrinum giganteum* var. *yunnanense*, a perennial herb in China with post-dispersal embryo growth. Seed Sci. Technol. 48, 303–314. doi: 10.15258/sst.2020.48.2.17

[B18] Li Q. Zhai W. Wei J. Jia Y. (2023a). Rice lipid transfer protein, OsLTPL23, controls seed germination by regulating starch-sugar conversion and ABA homeostasis. Front. Genet. 14. doi: 10.3389/fgene.2023.1111318, PMID: 36726806 PMC9885049

[B19] Liu J. Hasanuzzaman M. Wen H. Zhang J. Peng T. Sun H.. (2019). High temperature and drought stress cause abscisic acid and reactive oxygen species accumulation and suppress seed germination growth in rice. Protoplasma 256, 1217–1227. doi: 10.1007/s00709-019-01354-6, PMID: 31001689

[B20] Liu B. Lin R. Jiang Y. Jiang S. Xiong Y. Lian H.. (2020). Transcriptome analysis and identification of genes associated with starch metabolism in castanea henryi seed (Fagaceae). Int. J. Mol. Sci. 21, 1431. doi: 10.3390/ijms21041431, PMID: 32093295 PMC7073145

[B21] Luang S. Cho J.-I. Mahong B. Opassiri R. Akiyama T. Phasai K.. (2013). Rice os9BGlu31 is a transglucosidase with the capacity to equilibrate phenylpropanoid, flavonoid, and phytohormone glycoconjugates *. J. Biol. Chem. 288, 10111–10123. doi: 10.1074/jbc.M112.423533, PMID: 23430256 PMC3617254

[B22] Ma J. He R. Wei Y. (2022). China -Mother of Gardens, in the Twenty-first Century (Beijing, China: China Forestry Publishing House). Available online at: https://www.zhangqiaokeyan.com/book-cn/081504401712.html.

[B23] Mangrauthia S. K. Agarwal S. Sailaja B. Sarla N. Voleti S. R. (2016). Transcriptome Analysis of Oryza sativa (Rice) Seed Germination at High Temperature Shows Dynamics of Genome Expression Associated with Hormones Signalling and Abiotic Stress Pathways. Trop. Plant Biol. 9, 215–228. doi: 10.1007/s12042-016-9170-7

[B24] Meng A. Wen D. Zhang C. (2022). Dynamic changes in seed germination under low-temperature stress in maize. Int. J. Mol. Sci. 23, 5495. doi: 10.3390/ijms23105495, PMID: 35628306 PMC9141190

[B25] Nakashima K. Fujita Y. Kanamori N. Katagiri T. Umezawa T. Kidokoro S.. (2009). Three arabidopsis SnRK2 protein kinases, SRK2D/SnRK2.2, SRK2E/SnRK2.6/OST1 and SRK2I/SnRK2.3, involved in ABA signaling are essential for the control of seed development and dormancy. Plant Cell Physiol. 50, 1345–1363. doi: 10.1093/pcp/pcp083, PMID: 19541597

[B26] Née G. Xiang Y. Soppe W. J. (2017). The release of dormancy, a wake-up call for seeds to germinate. Curr. Opin. Plant Biol. 35, 8–14. doi: 10.1016/j.pbi.2016.09.002, PMID: 27710774

[B27] Nishimura N. Tsuchiya W. Moresco J. J. Hayashi Y. Satoh K. Kaiwa N.. (2018). Control of seed dormancy and germination by DOG1-AHG1 PP2C phosphatase complex via binding to heme. Nat. Commun. 9, 2132. doi: 10.1038/s41467-018-04437-9, PMID: 29875377 PMC5989226

[B28] Nonogaki H. (2020). A repressor complex silencing ABA signaling in seeds? J. Exp. Bot. 71, 2847–2853. doi: 10.1093/jxb/eraa062, PMID: 32004374

[B29] O’Lone C. E. Juhász A. Nye-Wood M. Moody D. Dunn H. Ral J.-P.. (2024). Advancing Sustainable Malting Practices: Aquaporins as Potential Breeding Targets for Improved Water Uptake during Controlled Germination of Barley (*Hordeum vulgare* L.). J. Agric. Food Chem. 72, 10149–10161. doi: 10.1021/acs.jafc.4c00884, PMID: 38635353 PMC11066872

[B30] Penfield S. MacGregor D. R. (2016). Effects of environmental variation during seed production on seed dormancy and germination. J. Exp. Bot. 68, 819–825. doi: 10.1093/jxb/erw436, PMID: 27940467

[B31] Phartyal S. S. Kondo T. Baskin C. C. Baskin J. M. (2012). Seed dormancy and germination in the giant Himalayan lily (*Cardiocrinum giganteum* var. *giganteum*): an assessment of its potential for naturalization in northern Japan. Ecol. Res. 27, 677–690. doi: 10.1007/s11284-012-0940-x

[B32] Quan C. Tang D. Jiang J. Zhu Z. (2025). Transcriptome sequencing reveals molecular mechanism of seed dormancy release of *Zanthoxylum nitidum* . China J. Chin. Materia Med. 50, 102–110. doi: 10.19540/j.cnki.cjcmm.20241014.103, PMID: 39929651

[B33] Reed R. C. Bradford K. J. Khanday I. (2022). Seed germination and vigor: ensuring crop sustainability in a changing climate. Heredity 128, 450–459. doi: 10.1038/s41437-022-00497-2, PMID: 35013549 PMC9177656

[B34] Ren X.-X. Xue J.-Q. Wang S.-L. Xue Y.-Q. Zhang P. Jiang H.-D.. (2018). Proteomic analysis of tree peony (Paeonia ostii ‘Feng Dan’) seed germination affected by low temperature. J. Plant Physiol. 224–225, 56–67. doi: 10.1016/j.jplph.2017.12.016, PMID: 29597068

[B35] Shao Z. Liu N. Wang W. Zhu L. (2022). β-Glucosidases as dominant dose-dependent regulators of *Oryza sativa* L. @ in response to typical organic pollutant exposures. Environ. pollut. 309, 119709. doi: 10.1016/j.envpol.2022.119709, PMID: 35841992

[B36] Shou J.-W. Zhang R.-R. Wu H.-Y. Xia X. Nie H. Jiang R.-W.. (2018). Isolation of novel biflavonoids from *Cardiocrinum giganteum* seeds and characterization of their antitussive activities. J. Ethnopharmacology 222, 171–176. doi: 10.1016/j.jep.2018.05.003, PMID: 29738848

[B37] Springthorpe V. Penfield S. (2015). Flowering time and seed dormancy control use external coincidence to generate life history strategy. eLife 4, e05557. doi: 10.7554/eLife.05557, PMID: 25824056 PMC4378508

[B38] Storey J. D. (2003). The positive false discovery rate: a Bayesian interpretation and the q-value. Ann. Stat 31, 2013–2035. doi: 10.1214/aos/1074290335

[B39] Sun C. Fang J. Xu X. (2000). Studies on the medicinal plant seed germination of lily family (Liliaceae). Chin. T raditional Herbal Drugs 31, 49–51.

[B40] Suriyasak C. Oyama Y. Ishida T. Mashiguchi K. Yamaguchi S. Hamaoka N.. (2020). Mechanism of delayed seed germination caused by high temperature during grain filling in rice (Oryza sativa L.). Sci. Rep. 10, 17378. doi: 10.1038/s41598-020-74281-9, PMID: 33060675 PMC7562956

[B41] Toh S. Imamura A. Watanabe A. Nakabayashi K. Okamoto M. Jikumaru Y.. (2008). High temperature-induced abscisic acid biosynthesis and its role in the inhibition of gibberellin action in arabidopsis seeds. Plant Physiol. 146, 1368–1385. doi: 10.1104/pp.107.113738, PMID: 18162586 PMC2259091

[B42] Tong Y. Liu S.-Y. Yi S.-C. Qiu Z.-X. Wang Y.-H. Zeng D.-Q.. (2021). Bruceine D, the main active ingredient of *Brucea javanica* (L.) residue inhibits the germination of *Bidens pilosa* L. seeds by suppressing phenylpropanoid biosynthesis. Ind. Crops Products 172, 114079. doi: 10.1016/j.indcrop.2021.114079

[B43] Umezawa T. Sugiyama N. Mizoguchi M. Hayashi S. Myouga F. Yamaguchi-Shinozaki K.. (2009). Type 2C protein phosphatases directly regulate abscisic acid-activated protein kinases in *Arabidopsis* . Proc. Natl. Acad. Sci. United States America 106, 17588–17593. doi: 10.1073/pnas.0907095106, PMID: 19805022 PMC2754379

[B44] Umezawa T. Yoshida R. Maruyama K. Yamaguchi-Shinozaki K. Shinozaki K. (2004). SRK2C, a SNF1-related protein kinase 2, improves drought tolerance by controlling stress-responsive gene expression in *Arabidopsis thaliana* . Proc. Natl. Acad. Sci. United States America 101, 17306–17311. doi: 10.1073/pnas.0407758101, PMID: 15561775 PMC535404

[B45] Wang H. Xu T. Li Y. Gao R. Tao X. Song J.. (2024). Comparative transcriptome analysis reveals the potential mechanism of GA3-induced dormancy release in Suaeda glauca black seeds. Front. Plant Sci. 15. doi: 10.3389/fpls.2024.1354141, PMID: 38919815 PMC11197467

[B46] Wang X. Zhang X. (2010). DEGseq: an R package for identifying differentially expressed genes from RNA-seq data. Bioinformatics 26, 136–138. doi: 10.1093/bioinformatics/btp612, PMID: 19855105

[B47] Wang X. Zhou W. Lu Z. Ouyang Y. O C. S. Yao J. (2015). A lipid transfer protein, OsLTPL36, is essential for seed development and seed quality in rice. Plant Sci. 239, 200–208. doi: 10.1016/j.plantsci.2015.07.016, PMID: 26398804

[B48] Winter H. Huber J. L. Huber S. C. (1997). Membrane association of sucrose synthase: changes during the graviresponse and possible control by protein phosphorylation. FEBS Lett. 420, 151–155. doi: 10.1016/S0014-5793(97)01506-8, PMID: 9459300

[B49] Yan M. Xue C. Xiong Y. Meng X. Li B. Shen R.. (2020). Proteomic dissection of the similar and different responses of wheat to drought, salinity and submergence during seed germination. J. Proteomics 220, 103756. doi: 10.1016/j.jprot.2020.103756, PMID: 32201361

[B50] Yang L.-E. Peng D.-L. Li Z.-M. Huang L. Yang J. Sun H. (2020). Cold stratification, temperature, light, GA3, and KNO3 effects on seed germination of *Primula beesiana* from Yunnan, China. Plant Diversity 42, 168–173. doi: 10.1016/j.pld.2020.01.003, PMID: 32695949 PMC7361177

[B51] Zaghdoud C. Ollio I. Solano C. J. Ochoa J. Suardiaz J. Fernández J. A.. (2023). Red LED Light Improves Pepper (Capsicum annuum L.) Seed Radicle Emergence and Growth through the Modulation of Aquaporins, Hormone Homeostasis, and Metabolite Remobilization. Int. J. Mol. Sci. 24, 4779. doi: 10.3390/ijms24054779, PMID: 36902208 PMC10002511

[B52] Zhang Y. Li D. Dirk L. M. A. Downie A. B. Zhao T. (2021). ZmAGA1 Hydrolyzes RFOs Late during the Lag Phase of Seed Germination, Shifting Sugar Metabolism toward Seed Germination Over Seed Aging Tolerance. J. Agric. Food Chem. 69, 11606–11615. doi: 10.1021/acs.jafc.1c03677, PMID: 34553917

[B53] Zhang M. Rana S. Li C. Zhang X. Tian K. Liu Z.. (2024). Transcriptome analysis reveals the role of temperature in seed germination of Idesia polycarpa Maxim through the integration of phytohormones and sugar metabolism. Braz. J. Bot. 47, 963–979. doi: 10.1007/s40415-024-01027-6

[B54] Zhao H. Yin C. Ma B. Chen S. Zhang J. (2021). Ethylene signaling in rice and *Arabidopsis*: New regulators and mechanisms. J. Integr. Plant Biol. 63, 102–125. doi: 10.1111/jipb.13028, PMID: 33095478

[B55] Zhao M. Zhang H. Yan H. Qiu L. Baskin C. C. (2018). Mobilization and role of starch, protein, and fat reserves during seed germination of six wild grassland species. Front. Plant Sci. 9. doi: 10.3389/fpls.2018.00234, PMID: 29535748 PMC5835038

[B56] Zhao Z. Huang L. Huang X. Liao J. Zeng G. Liu D. (2025). Assembly and comparative analysis of the complete mitochondrial genome of Cardiocrinum giganteum: a primitive Liliaceae group with significant scientific research value. BMC Genomics 26, 602. doi: 10.1186/s12864-025-11817-1, PMID: 40597596 PMC12211521

