# Supplementary material for: Comparative transcriptome analysis reveal gene regulation of dormancy release in Cardiocrinum giganteum seeds induced by temperature
Source: Front Plant Sci. 2025 Jul 17;16:1591781. doi: 10.3389/fpls.2025.1591781 (PMC12310627; doi:10.3389/fpls.2025.1591781)
Supplement: Supplementary file 1 [file Table1.docx]

**TABLE S1** Significant up-regulated genes during seed stratification of *C. giganteum*

| **Gene_id** | **Pathway** | **Description** | **0d** | **30d** | **60d** | **90d** | **120d** | **130d** |
| --- | --- | --- | --- | --- | --- | --- | --- | --- |
| Cluster-62345.16743 | Biosynthetic process | ADP-glucose pyrophosphorylase large subunit | 0.03 | 0.42 | 0.7333 | 13.277 | 32.617 | 46.077 |
| Cluster-62345.51535 | | ADP-glucose pyrophosphorylase small subunit | 0.08 | 0.5667 | 0.71 | 16.57 | 28.42 | 63.253 |
| Cluster-62345.15705 | Carbohydrate metabolic process | Glucan endo-1,3-beta-glucosidase 9 | 0.0367 | 1.47 | 1.52 | 12.553 | 24.527 | 22.423 |
| Cluster-62345.17126 | | Alpha-xylosidase 1 | 0.85 | 8.6267 | 10.35 | 37.3 | 43.863 | 95.737 |
| Cluster-62345.17679 | | Aldose 1-epimerase | 0.0867 | 0.3 | 1.26 | 61.03 | 138.74 | 107.59 |
| Cluster-62345.47720 | | Glucan endo-1,3-beta-glucosidase 12 | 0.14 | 3.0867 | 2.5167 | 63.657 | 88.177 | 122.26 |
| Cluster-62345.50561 | | Beta-D-xylosidase 7 | 0.3033 | 1.1367 | 1.6233 | 14.113 | 25.223 | 46.247 |
| Cluster-62345.16067 | | Polygalacturonase-like | 0.28 | 1.7433 | 1.52 | 13.073 | 15.763 | 28.833 |
| Cluster-62345.50027 | | GBSS1 | 0.8667 | 5.6267 | 8.34 | 151.27 | 220.96 | 419.32 |
| Cluster-62345.16277 | DNA replication | 21 kDa protein-like | 1.33 | 6.5467 | 11.773 | 37.783 | 72.743 | 70.16 |
| Cluster-62345.17413 | Hydrolase activity | Non-specific phospholipase C2 | 0.01 | 1.2667 | 1.4733 | 6.7533 | 9.1967 | 14.773 |
| Cluster-62345.51274 | | Purple acid phosphatase 1 | 0.6133 | 2.7633 | 2.5733 | 13.307 | 14.82 | 36.237 |
| Cluster-62345.12109 | Metabolic process | Tubulin | 0.0633 | 4.3633 | 2.6433 | 14.563 | 18.47 | 27.787 |
| Cluster-62345.27432 | | 4-coumarate--CoA ligase | 0.5767 | 2.08 | 1.95 | 4.11 | 3.2133 | 7.2867 |
| Cluster-62345.42132 | | Pentatricopeptide repeat-containing protein | 2.6333 | 6.37 | 6.1367 | 19.907 | 17.413 | 35.807 |
| Cluster-62345.50115 | | Methyltransferase PMT17 | 1.6167 | 4.37 | 5.73 | 20.103 | 34.873 | 37.527 |
| Cluster-62345.17189 | | Pectinesterase 68 | 0.0133 | 0.5867 | 0.5567 | 6.84 | 9.9933 | 21.043 |
| Cluster-62345.28813 | | Peroxidase | 44.047 | 113.29 | 97.187 | 368.95 | 580.47 | 1388.2 |
| Cluster-62345.17181 | Metal ion binding | Zinc finger protein 4 | 0.4367 | 4.9767 | 6.43 | 17.607 | 38.457 | 43.32 |
| Cluster-62345.16687 | Oxidation-reduction process | L-ascorbate oxidase homolog | 0.1567 | 0.2467 | 1.1033 | 29.657 | 50.977 | 60.933 |
| Cluster-62345.17199 | | CYP736A12 | 0.1367 | 0.1933 | 1.1233 | 65.49 | 108.38 | 174.9 |
| Cluster-62345.50124 | | Linoleate 9S-lipoxygenase | 0.46 | 2.32 | 4.21 | 30.53 | 227.61 | 426.43 |
| Cluster-62345.15506 | | CYP450 family protein | 0.03 | 0.14 | 0.62 | 27.64 | 35.167 | 63.077 |
| Cluster-62345.55998 | Photosynthesis | Photosystem II oxygen evolving complex protein 1 precursor | 0.2467 | 3.5133 | 3.7433 | 11.74 | 35.483 | 62.087 |
| Cluster-62345.10044 | | PSBP domain-containing protein 3 | 0.33 | 1.3733 | 2.4433 | 7.92 | 7.8733 | 20.29 |
| Cluster-62345.11927 | Plant hormone signal transduction | Ethylene receptor 2-like | 0.0267 | 0.52 | 0.7967 | 2.27 | 2.2033 | 8.7467 |
| Cluster-62345.17323 | | Gibberellin-regulated protein 6 | 0.0633 | 0.92 | 1.6567 | 97.893 | 518.04 | 558.61 |
| Cluster-62345.25404 | | Auxin-responsive protein IAA6-like | 0.4733 | 0.8067 | 1.28 | 3.3767 | 2.95 | 7.9967 |
| Cluster-62345.49899 | | CYP90B1 | 2.7 | 7.3167 | 7.6 | 189.4 | 575.23 | 549.31 |
| Cluster-62345.51180 | | Gibberellin-regulated protein 9 isoform X2 | 0.01 | 0.4733 | 0.4433 | 8.19 | 14.837 | 25.893 |
| Cluster-62345.15891 | Protein phosphorylation | Serine/threonine-protein kinase D6PK | 0.2167 | 0.85 | 1.59 | 15.613 | 13.877 | 34.823 |
| Cluster-62345.16434 | | Serine/threonine-protein kinase BAM1 | 0.1767 | 1.48 | 1.66 | 8.7733 | 10.127 | 17.01 |
| Cluster-62345.13420 | | PINOID 2-like | 0.1133 | 3.2633 | 2.63 | 8.03 | 19.257 | 46.38 |
| Cluster-62345.16582 | Proteolysis | Aspartic protease in guard cell 2-like | 0.03 | 0.7433 | 0.9 | 10.47 | 18.92 | 38.573 |
| Cluster-62345.19091 | | Subtilisin-like protease SBT2.5 | 2.2267 | 4.35 | 4.93 | 23.123 | 26.337 | 41.46 |
| Cluster-62345.16786 | Transcription regulation | bHLH94-like | 0.09 | 1.1633 | 1.1433 | 9.8433 | 17.75 | 34.22 |
| Cluster-62345.16927 | | bHLH94 | 0.0967 | 1.3533 | 1.9433 | 15.803 | 28.05 | 30.277 |
| Cluster-62345.16930 | | Cyclin-D1-1-like | 0.4067 | 3.8533 | 3.2533 | 25.877 | 45.8 | 48.237 |
| Cluster-62345.23693 | | PCF5-like | 4.6433 | 8.0333 | 11.62 | 50.647 | 66.3 | 208.29 |
| Cluster-62345.14937 |  | Homeobox-leucine zipper protein HAT5 | 0.3967 | 1.86 | 3.4333 | 10.657 | 22.767 | 39.153 |
| Cluster-62345.14961 | | Zinc-finger homeodomain protein 6-like | 0.0267 | 1.24 | 1.3967 | 4.8133 | 6.43 | 8.9067 |
| Cluster-62345.15153 | | Zinc-finger homeodomain protein 4-like | 0.0233 | 0.5133 | 1.2267 | 6.2367 | 7.4067 | 18.52 |
| Cluster-62345.17149 | | Homeobox-leucine zipper protein HOX21 | 0.3967 | 0.5633 | 2.29 | 17.023 | 17.37 | 31.23 |
| Cluster-62345.17225 | | GATA transcription factor | 0.8333 | 6.5167 | 7.0967 | 35.643 | 223.14 | 252.2 |
| Cluster-62345.17849 | | Subtilisin-like protease SBT1.7 | 3.1533 | 10.833 | 17.797 | 171.27 | 233.89 | 356.89 |
| Cluster-62345.16820 | Ribosome biogenesis | Protein TSS | 0.2633 | 0.93 | 1.1533 | 3.97 | 4.94 | 12.443 |
| Cluster-62345.23908 | Transport | Heavy metal-associated isoprenylated plant protein 3-like | 3.8767 | 25.463 | 26.1 | 236.82 | 273.98 | 611.42 |
| Cluster-62345.17020 | | Aquaporin PIP | 0.3233 | 2.4033 | 3.7467 | 123.96 | 111.97 | 238.48 |
| Cluster-62345.17332 | | GDSL esterase/lipase | 1.5467 | 4.4233 | 5.92 | 60.84 | 80.747 | 128.82 |
| Cluster-62345.42060 | | Aquaporin PIP | 32.51 | 66.687 | 84.093 | 222 | 317.45 | 393.98 |
| Cluster-62345.49973 | | Aquaporin TIP | 6.2067 | 56.66 | 65.71 | 719.81 | 1504.3 | 1822.9 |
| Cluster-62345.14262 | | Epidermis-specific secreted glycoprotein EP1 | 0.0167 | 0.4367 | 1.47 | 10.657 | 16.633 | 41.31 |
| Cluster-62345.50153 | | Protein NRT1/ PTR FAMILY 4.2 | 0.2933 | 1.6733 | 2.4867 | 14.323 | 27.587 | 37.893 |
| Cluster-62345.15252 | Uncharacterized | Root phototropism protein 3 | 0.2333 | 2.0133 | 2.43 | 6.9167 | 10.68 | 16.633 |
| Cluster-62345.16479 | | S-adenosylmethionine carrier 1 | 1.87 | 4.2933 | 5.5767 | 13.893 | 12.537 | 26.89 |
| Cluster-62345.18000 | | Uncharacterized protein | 1.05 | 3.9467 | 3.75 | 9.0667 | 12.517 | 18.303 |
| Cluster-62345.18067 | | Uncharacterized protein | 0.4167 | 2.6933 | 3.1467 | 79.833 | 110.38 | 181.94 |
| Cluster-62345.30298 | | Uncharacterized protein | 1.05 | 3.6533 | 3.67 | 22.667 | 26.57 | 47.487 |
| Cluster-62345.33754 | | uncharacterized protein | 1.87 | 27.67 | 29.51 | 281.04 | 264.64 | 660.05 |
| Cluster-62345.49903 | | Ribulose-bisphosphate carboxylase small chain | 0.7267 | 5.6833 | 7.91 | 170.93 | 678.28 | 1817.4 |
| Cluster-62345.50138 | | 14 kDa proline-rich protein | 0.0367 | 5.58 | 8.01 | 43.337 | 59.44 | 144.96 |
| Cluster-62345.50624 | | Uncharacterized protein | 0 | 0.1633 | 0.81 | 7.0467 | 11.733 | 35.44 |
| Cluster-62345.51898 | | Leucine-rich repeat-containing protein | 0.0833 | 1.2367 | 1.3533 | 4.6767 | 5.76 | 9.41 |
| Cluster-62345.9910 | | Uncharacterized protein | 0.0433 | 0.2833 | 0.7067 | 1.83 | 1.84 | 3.6067 |
| Cluster-62345.16693 | | Snakin-2-like | 0.47 | 4.8267 | 10.867 | 53.48 | 105.46 | 140.11 |
| Cluster-62345.8883 | | Threonine--tRNA ligase | 0.8567 | 2.39 | 2.5933 | 5.17 | 5.4567 | 9.2433 |
